# α-Glucosidase Inhibitors: Diphenyl Ethers and Phenolic Bisabolane Sesquiterpenoids from the Mangrove Endophytic Fungus *Aspergillus flavus* QQSG-3

**DOI:** 10.3390/md16090307

**Published:** 2018-09-01

**Authors:** Yingnan Wu, Yan Chen, Xishan Huang, Yahong Pan, Zhaoming Liu, Tao Yan, Wenhao Cao, Zhigang She

**Affiliations:** 1 School of Chemistry, Sun Yat-Sen University, Guangzhou 510275, China; wuyn3@mail2.sysu.edu.cn (Y.W.); huangxsh9@mail.sysu.edu.cn (X.H.); pan16a@126.com (Y.P.); 2 South China Sea Bio-Resource Exploitation and Utilization Collaborative Innovation Center, School of Marine Sciences, Sun Yat-Sen University, Guangzhou 510006, China; chenyan27@mail2.sysu.edu.cn; 3 State Key Laboratory of Applied Microbiology, Southern China, Guangdong Institute of Microbiology, Guangzhou 510075, China; 4 CAS Key Laboratory of Tropical Marine Bio-Resources and Ecology, Guangdong Key Laboratory of Marine Materia, RNAM Center for Marine Microbiology, South China Sea Institute of Oceanology, Chinese Academy of Sciences, Guangzhou 510301, China; yantao@scsio.ac.cn (T.Y.); chromo@163.com (W.C.)

**Keywords:** α-glucosidase inhibitors, mangrove endophytic fungus, *Aspergillus flavus*

## Abstract

Two new diphenyl ethers (**1** and **2**) and four new phenolic bisabolane sesquiterpenoids (**3**–**6**), together with five known related derivatives, were isolated from the culture of the endophytic fungus *Aspergillus flavus* QQSG-3 obtained from a fresh branch of *Kandelia obobata*, which was collected from Huizhou city in the province of Guangdong, China. The structures of compounds **1**–**6** were determined by analyzing NMR and HRESIMS data. The absolute configurations of **5** and **6** were assigned by comparing their experimental ECD spectra with those reported for similar compounds in the literature. All isolates were evaluated for their α-glucosidase inhibitory activity, of which compounds **3**, **5**, **10**, and **11** showed strong inhibitory effects with IC_50_ values in the range of 1.5–4.5 μM.

## 1. Introduction

Chemical investigations on mangrove endophytic fungi have generated considerable attention from natural product researchers due to their unique ecosystem, which leads to the isolation of secondary metabolites with diverse structures and excellent biological activities. The genus *Aspergillus*, a ubiquitous fungus, is recognized as a rich source of biomolecules for constructing novel skeletons for drug discovery, including terpenoids [1,2,3], cyclopeptides [4,5], alkaloids [6], butenolides [7], coumarins [8], and quinones [9]. As a class of terpenoids, their diverse biological activities gave rise to phenolic bisabolane sesquiterpenoids getting more attention, including antibacterial activities [10,11], acetylcholinesterase inhibition [12], cytotoxicity [13], and antioxidant activities [14].

**Figure 1:**
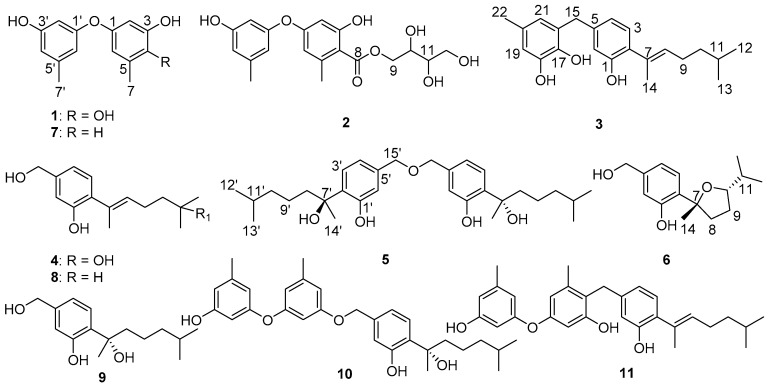
Chemical structures of compounds **1**–**11**.

As part of our efforts to discover potent and new α-glucosidase inhibitors [15,16,17,18], *Aspergillus flavus* QQSG-3 was chemically investigated, which was isolated from a fresh branch of *Kandelia obovata* collected from Huizhou, Guangdong, China. The EtOAc extract afforded two new diphenyl ethers (**1** and **2**), four new phenolic bisabolane sesquiterpenoids (**3**–**6**), and five known compounds (**7**–**11**) (Figure 1). The α-glucosidase inhibitory activities of all isolates were tested. Compounds **3**, **5**, **10**, and **11** exhibited strong inhibitory effects compared to the positive control acarbose. In this paper, we describe the purification, structure elucidation, and bioactivities of these compounds.

## 2. Results

**Figure 2:**
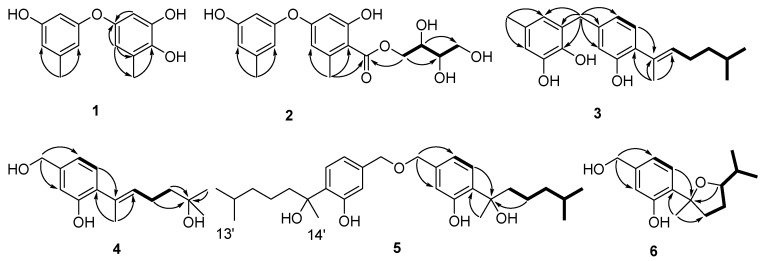
Key COSY (bold line) and HMBC (arrow) correlations of compounds **1**–**6**.

Compound **1** was obtained as a colorless oil. The molecular formula was deduced to be C_14_H_14_O_4_ based on the protonated molecular ion peak at *m*/*z* 247.0962 ([M + H]^+^, calcd. for 247.0964) from HRESIMS, indicating eight degrees of unsaturation. The ^1^H NMR (Table 1) showed five aromatic protons (*δ*_H_ 6.43 (1H, d, *J* = 2.6 Hz), 6.41 (1H, d, *J* = 2.6 Hz), 6.22 (1H, dd, *J* = 3.6, 2.1 Hz), 6.34 (1H, brs), and 6.34 (1H, brs)), and two methyl groups (*δ*_H_ 2.24 (3H, s) and 2.22 (3H, s)). The ^13^C NMR spectrum (Table 1) displayed 12 aromatic carbons (*δ*_C_ 159.6, 156.5, 149.4, 144.0, 141.0, 138.5, 125.5, 114.1, 111.1, 110.5, 105.5, and 102.3) and two methyl carbons (*δ*_C_ 21.6, 15.8). The 1D NMR data compared to the known compound cordyol C [19] indicated the same diphenyl ether skeleton was present in compound **1**. The main difference was the obvious upfield shift of H-2 (*δ*_H_ 6.43) and C-7 (*δ*_C_ 15.8), which suggested the different substituted location of the hydroxyl group (4-OH in **1** and 2-OH in cordyol C). Combined with HMBC correlations of H-6/C-1, C-2, C-4, C-5, and C-7; and H-2/C-1, C-3, and C-4 (Figure 2), the 2,3,5-trihydroxytoluene unit was established. The 3,5-dihydroxytoluene unit was supposed based on the ^1^H NMR signals of *δ*_H_ 6.22 (dd, *J* = 3.6, 2.1 Hz, H-2′), 6.34 (brs, H-4′), and 6.34 (brs, H-6′), which was further confirmed by the correlations from H_3_-7′ to C-3′, C-4′, and C-5′; from H-2′/H-4′ to C-3′; and from H-2′/H-6′ to C-1′. Thus, compound **1** was identified as shown in Figure 1.

Compound **2** was isolated as a colorless oil. Its molecular formula was established as C_19_H_21_O_8_ on the basis of HRESIMS data (*m*/*z* 377.1245 [M − H]^−^ (calcd. for 377.1242)). Compound **2** contained two structural moieties (a diphenyl ether moiety similar to **1** and an erythritol moiety) deduced from the analysis of 1D and 2D NMR signals. The weak four-bond HMBC correlations from H-2 and H-6 to C-8 (Figure 2) revealed the replacement of OH-4 in **1** with the carbonyl carbon, C-8 (*δ*_C_ 172.4). This was further confirmed by the downfield shift of C-3 (*δ*_C_ 164.9) and upfield shift of C-4 (*δ*_C_ 109.9) caused by the electron-donating conjugative effect of C-8. The erythritol moiety was confirmed by the HSQC data and COSY correlations from H_2_-9 to H-10, H-10 to H-11, and H-11 to H_2_-12 (Figure 2). The moiety has a linkage with C-8 that was verified by the key correlation from H_2_-9 to C-8 in HMBC. However, acid hydrolysis of compound **2** could not be performed due to its limited quantity, and the absolute configuration was not assigned. 

Compound **3** was a pale yellow oil with the molecular formula C_22_H_28_O_3_ established from HRESIMS at *m*/*z* 339.1963 [M − H]^−^ (calcd. for 339.1965). The ^1^H NMR spectrum displayed characteristic aromatic protons of 1,2,4-trisubstituted benzene (*δ*_H_ 6.88 (d, *J* = 7.7 Hz), 6.63 (dd, *J* = 7.7, 1.4 Hz), 6.60 (d, *J* = 1.4 Hz)), 1,2,3,5-tetrasubstituted benzene (*δ*H 6.47 (d, *J* = 1.3 Hz), 6.34 (dd, *J* = 1.3 Hz)), and a trisubstituted double bond (*δ*_H_ 5.38 (td, *J* = 7.2, 1.3 Hz)) (Table 2). The key HMBC correlations from H_2_-15 to C-4, C-5, C-6, C-16, C-17, and C-21 revealed that two benzene moieties were connected to each other via a methylene (C-15) between C-5 and C-20 (Figure 2). The ^1^H–^1^H COSY cross peaks from H-8 to H-13 established the only spin coupling system, which could be constructed to the side chain based on the HMBC correlations between H_3_-14 to C-2, C-7, and C-8 (Figure 2). H_2_-9 displayed a cross-peak with the olefinic proton H-8, which, as well as the correlations from H_3_-14 to C-7 and C-8 in HMBC, established a 6-methyl-2-heptenyl unit. This unit was attached at C-2 on the basis of the HMBC correlations of H-8/C-2. The *E*-geometry for the trisubstituted double bond was deduced from the signal enhancement of H_2_-9 upon irradiation of H_3_-14 in the NOEDIFF experiment [20] (Supporting Information Figures S21 and S22). Therefore, the configuration of **3** was determined.

Compound **4** was isolated as a colorless oil. Analysis of its HRESIMS data indicated compound **4** had the molecular formula C_15_H_22_O_3_, *m*/*z* 249.1498 [M − H]^−^ (calcd. for 249.1496). ^1^H and ^13^C NMR spectra were similar to those of compound **8**, except for the downfield shift for C-11 (*δ*_C_ 71.4). These data suggested that the hydroxyl had a linkage with C-11. This deduction was further supported by the HMBC correlations from H_3_-12, H_2_-10, and H_2_-9 to C-11 (Figure 2). The *E*-geometry for the double bond was also deduced from the signal enhancement of H_2_-9 upon irradiation of H_3_-14 in the NOEDIFF experiment [20] (Supporting Information Figure S29). 

The molecular formula of compound **5** was established as C_30_H_46_O_5_ by the HRESIMS ion at *m*/*z* 485.3271 [M − H]^−^ (calcd. for 485.3272). The ^13^C NMR spectrum displayed fifteen carbon signals that were close to those of sydonol, **9**. The major difference was that the chemical shift of C-15 (C-15′) was changed from *δ*_C_ 64.8 in **9** (numbered as C-15) to *δ*_C_ 72.6 in compound **5** (Table 3). These data indicated that compound **5** was a dimeric analogue of sydonol **9**. Moreover, the HMBC correlation from H_2_-15 to C-15 (C-15′) also supported the dimer construction. The ECD spectrum showed one negative Cotton effect (CE) at 209 nm and two positive CEs near 227 and 279 nm (Supporting Information Figure S36), which were identical to that of peniciaculin B [21]. Thus, the absolute configuration of compound **5** was speculated to be 7*S*, 7′*S*, which is the same as peniciaculin B.

Compound **6** was isolated as a colorless oil and had the molecular formula of C_15_H_22_O_3_, determined by HRESIMS data *m*/*z* 249.1497 [M − H]^−^ (calcd. for 249.1496) with five degrees of unsaturation. The 1D NMR and HSQC data displayed three aromatic protons at *δ*_H_ 7.14 (1H, d, *J* = 7.9 Hz), 6.78 (1H, dd, *J* = 7.9, 1.1 Hz), and 6.75 (1H, d, *J* = 1.1 Hz); three methyl protons at *δ*_H_ 1.50 (3H, s), 1.03 (3H, d, *J* = 6.7 Hz), and 0.93 (3H, d, *J* = 6.7 Hz); three methylene protons at *δ*_H_ 4.50 (2H, s); 2.14 (1H, m), 2.40 (1H, m); and 1.71 (1H, m), 1.86 (1H, m); two methine protons at *δ*_H_ 1.75 (1H, m) and 3.65 (1H, m); six aromatic carbons at *δ*_C_ 155.8, 142.8, 132.0, 127.2, 119.0, and 116.0; three methyl carbons at *δ*_C_ 29.5, 19.5, and 18.9; three methylene carbons at *δ*_C_ 64.9, 39.5, and 29.7; two methine carbons at *δ*_C_ 86.4 and 34.4; and one oxygenated quaternary carbon at δ_C_ 87.2 (Table 2). The NMR data of **6** were similar to those of the known compound (7*R*,10*S*)-7,10-epoxysydonic acid [22], except for the absence of the carboxyl carbon in ^13^C NMR and the presence of additional hydroxymethyl protons, H_2_-15 (*δ*_H_ 4.50), in the ^1^H NMR, revealing that the carboxylic was reduced into a hydroxymethyl group in **6**. Analysis of the key correlations of H-10/C-7 in the HMBC data indicated that C-7 was linked to C-10 via an ether bond (Figure 2). C-14 and H-10 were oriented on the same side of the furan ring, which could be established based on the NOE correlations of H-10/H_3_-14. The absolute configuration of **6** was speculated as 7*R*,10*S* on the basis of the similar Cotton effects in the ECD spectrum compared (Supporting Information Figure S44) with that of (7*R*,10*S*)-7,10-epoxysydonic acid. 

In addition, the structures of diorcinol (**7**) [23], (*E*)-5-(hydroxymethyl)-2-(6′-methylhept-2’-en-2’-yl) phenol (**8**) [24], sydonol (**9**) [25], peniciaculin A (**10**) [21], and expansol D (**11**) [26] were identified by comparing their NMR data with those reported in the literature.

The isolated compounds (**1**–**11**) were evaluated for their inhibitory activities against α-glucosidase (Table 4). The results displayed that compounds **3**, **5**, **10**, and **11** were strong inhibitors with the IC_50_ values of 4.5, 3.1, 1.5, and 2.3 μM, respectively. Moreover, the activities of compounds **1** and **2** were better than that of acarbose (used as a positive control).

## 3. Experimental Section

### 3.1. General Experimental Procedures

Optical rotations were recorded in MeOH on an MCP 300 (Anton Paar, Shanghai, China) polarimeter at 25 °C. UV data were obtained on a PERSEE TU-1900 spectrophotometer (Persee, Beijing, China). IR spectra were measured in KBr on a Nicolet Nexus 670 spectrophotometer (Nicolet, Madison, WI, USA). CD data were measured on a J-810 spectropolarimeter (JASCO, Tokyo, Japan). The NMR spectra were performed on a Bruker Avance 500 spectrometer (^1^H/500 MHz, ^13^C/125 MHz, Bruker Bio Spin Corporation, Bellerica, MA, USA). All chemical shifts (*δ*) are given in ppm and coupling constants (*J*) are given in Hz. HRESIMS data were recorded on a Thermo Fisher Scientific Q-TOF mass spectrometer (Thermo Fisher Scientific, Waltham, MA, USA). Column chromatography (CC) was carried out on silica gel (200–300 mesh, Qingdao Marine Chemical Factory, Qingdao, China) and Sephadex LH-20 (Amersham Pharmacia, Piscataway, NJ, USA). Thin-layer chromatography (TLC) was performed on silica gel plates (Qingdao Huang Hai Chemical Group Co., G60, F-254, Qingdao, China). Phenomenex Luna (Phenomenex, Torrance, CA, USA) C_18_ column (250 × 10 mm, 5 μm) was used for semipreparative HPLC. 

### 3.2. Fungal Materials

The fungus QQSG-3 investigated in this study was isolated from a fresh branch of the mangrove plant *Kandelia obovata* collected from Huizhou in Guangdong Province, China, in August 2015. Upon analysis of ITS sequence data of rDNA, the strain was identified as *Aspergillus flavus*, which had 99% sequence identity to that of *Aspergillus flavus* (GenBank FJ011545.1). A voucher specimen has been deposited at the Guangdong Microbial Culture Center (patent depository number GDMCC 60380).

### 3.3. Extraction and Isolation

The fungus *Aspergillus flavus* QQSG-3 was grown in 60 1000-mL Erlenmeyer flasks at 27 °C for 28 days, containing autoclaved rice solid-substrate medium composed of 50 g rice and 50 mL 3‰ saline water. After incubation, the mycelia were extracted with EtOAc and the extract was concentrated to yield 11.3 g of residue under reduced pressure. Then, the residue was eluted by a gradient of petroleum ether/EtOAc from 9:1 to 0:10 on silica gel CC (480 × 110 mm) and divided into ten fractions (Fr.1–Fr.10). Fr.3 (812 mg) was further eluted on silica gel CC (280 × 20 mm) by 3:7 (petroleum ether/EtOAc) to give compound **8** (147 mg) and five fractions (Fr.3.1–Fr.3.5). Fr.3.1 (215 mg) was subjected to Sephadex LH-20 CC (300 × 25 mm) and eluted with MeOH to obtain compound **1** (3.8 mg), **7** (107 mg) and **9** (10.8 mg). Fr.3.4 (7 mg) was purified by semipreparative RP-HPLC (MeOH/H_2_O, 70/30; 1.0 mL/min) to afford compound **4** (1.5 mg, t_R_ = 17.5 min) and **6** (1.2 mg, t_R_ = 20.3 min). Fr.4 (78 mg) was eluted (by petroleum ether/EtOAc, 4:7) to obtain seven fractions (Fr.4.1–Fr.4.7). Fr.4.2 (3.8 mg) was applied to Sephadex LH-20 CC (300 × 25 mm) and was eluted with MeOH/CD_2_Cl_2_ (1:1) to yield compounds **2** (1.1 mg) and **5** (1.3 mg). Fr.4.5 (11.5 mg) was further purified by CC over silica gel (200 × 15 mm) using MeOH/CD_2_Cl_2_ (1:40) to furnish compound **3** (1.4 mg), **10** (3.1 mg), and **11** (2.5 mg), respectively.

Compound **1**: colorless oil; UV (MeOH) *λ*_max_ (log*ε*): 220 (4.23), 281 (3.47) nm; IR (KBr) *ν*_max_: 3371, 2934, 1614, 1476, 1307, 1139, 1046, 840 cm^−1^; ^1^H and ^13^C NMR data, see Table 1; HRESIMS *m*/*z* 247.0962 [M + H]^+^ (calcd. for 247.0964).
Compound **2**: colorless oil; UV (MeOH) *λ*_max_ (log*ε*): 216 (5.12), 264 (3.55), 305 (1.36) nm; IR (KBr) *ν*_max_: 3362, 2915, 1672, 1590, 1461, 1306, 1266, 1168 cm^−1^; ^1^H and ^13^C NMR data, see Table 1; HRESIMS *m*/*z* 377.1245 [M − H]^−^ (calcd. for 377.1242).
Compound **3**: pale yellow oil; UV (MeOH) *λ*_max_ (log*ε*): 213 (4.98), 285 (2.16) nm; IR (KBr) *ν*_max_: 3468, 2956, 1623, 1493, 1412, 1306, 1184 cm^−1^; ^1^H and ^13^C NMR data, see Table 2; HRESIMS *m/z* 339.1963 [M − H]^−^ (calcd. for 339.1965).
Compound **4**: colorless oil; UV (MeOH) *λ*_max_ (log*ε*): 233 (4.27), 285 (3.63) nm; IR (KBr) *ν*_max_: 3414, 2931, 1550, 1317, 846 cm^−1^; ^1^H and ^13^C NMR data, see Table 2; HRESIMS *m*/*z* 249.1498 [M − H]^−^ (calcd. for 249.1496).
Compound **5**: colorless oil; ${\left[\alpha \right]}_{D}^{25}$ + 3.2 (c 0.18, MeOH); UV (MeOH) *λ*_max_ (log*ε*): 203 (4.05), 221 (3.51), 280 (1.17) nm; IR (KBr) *ν*_max_: 3387, 2948, 1379, 1282, 772 cm^−1^; ECD *λ*_max_ (∆*ε*): 209 (−1.20), 227 (+1.29), 279 (+1.65); ^1^H and ^13^C NMR data, see Table 3; HRESIMS *m*/*z* 485.3271 [M − H]^−^ (calcd. for 485.3272).
Compound **6**: colorless oil; ${\left[\alpha \right]}_{D}^{25}$ + 22.3 (c 0.05, MeOH); UV (MeOH) *λ*_max_ (log*ε*): 202 (4.32), 218 (3.98), 279 (3.55) nm; IR (KBr) *ν*_max_: 3224, 2932, 1388, 1022, 872 cm^−1^; ECD *λ*_max_ (∆*ε*): 210 (−0.20), 222 (+1.94), 278 (+0.98); ^1^H and ^13^C NMR data, see Table 2; HRESIMS *m/z* 249.1497 [M − H]^−^ (calcd. for 249.1496).

### 3.4. Biological Assays

α-Glucosidase was assayed according to standard procedures [27,28]. Compounds **1**–**11** and acarbose (positive control) were dissolved in DMSO, and enzyme (0.4 units/mL) and substrate (p-nitrophenyl-α-glucopyranoside, 5 mM) were dissolved in phosphate buffer solution (PBS, 100 mM, pH 7). Ten microliters of testing materials (in triplicate) were incubated for 10 min with 20 μL of enzyme stock solution and 60 μL of PBS. After incubation, 10 μL of substrate was added and incubated for 20 min at 37 °C. Absorbance at 405 nm was then determined.

The inhibitory activity of the isolates was determined as a percentage in comparison to a blank (DMSO) according to the following equation:

$$\%\alpha \mathrm{GHY}=\left(1-\frac{A_{405}t}{A_{405}c}\right)\times 100\%$$

where %αGHY is the percentage of inhibition, A_405_t is the corrected absorbance of the compound under testing (A_405end_ − A_405initial_), and A_405_c is the absorbance of the blank (A_405endblank_ − A_405initialblank_). The IC_50_ values of compounds were calculated by the nonlinear regression analysis.

## 4. Conclusions

In summary, two new diphenyl ether derivatives, **1** and **2**, and four new phenolic bisabolane sesquiterpenoids, **3**–**6**, along with five known compounds were isolated from the mangrove endophytic fungus *Aspergillus flavus* QQSG-3. Bioactivity assays displayed that phenolic bisabolane sesquiterpenoid derivatives **3**, **5**, **10**, and **11** exhibited strong inhibitory activity against α-glucosidase, suggesting that they might have potential to be developed as α-glucosidase inhibitors. This is the first report of α-glucosidase inhibitory activity of diphenyl ethers and phenolic bisabolane sesquiterpenoids. Our findings enrich the diversity of their family and bioactivities.

## Figures and Tables

**Table 1:** ^1^H and ^13^C NMR spectroscopic data (500/100 MHz) for **1** and **2** ^a^.

No.	1		2	
	*δ*_C_	*δ*_H_	*δ*_C_	*δ*_H_
1	149.4		163.6	
2	105.5	6.43, d (2.6)	103.7	6.23, s
3	144.0		164.9	
4	138.4		109.9	
5	125		144.6	
6	114.1	6.41, d (2.6)	113.1	6.35, s
7	15.8	2.22, s	24.0	2.53, s
8			172.4	
9			68.4	4.40, dd (6.6, 11.6) 4.62, dd (2.8, 11.6)
10			71.1	3.89, td (2.8, 6.8)
11			73.7	3.63, m
12			64.6	3.65, dd (5.3, 8.0) 3.77, dd (5.7, 7.7)
1′	159.6		159.9	
2′	102.3	6.22, dd (3.6, 2.1)	105.7	6.27, s
3′	156.5		157.4	
4′	111.1	6.34, brs	113.6	6.47, s
5′	141.1		142.2	
6′	110.5	6.34, brs	113.4	6.35, s
7′	21.6	2.24, s	21.5	2.26, s

^a^*δ* in ppm, *J* in Hz, **1** in CDCl_3_, **2** in methanol-*d*_4_.

**Table 2:** ^1^H and ^13^C NMR spectroscopic data (500/100 MHz) for **3**, **4**, and **6**^a^.

No.	3		4		6	
	*δ*_C_	*δ*_H_	*δ*_C_	*δ*_H_	*δ*_C_	*δ*_H_
1	154.9		155.2		155.8	
2	131.6		133.2		132.0	
3	130.5	6.88, d (7.7)	130.3	6.99, d (7.7)	127.2	7.14, d (7.9)
4	121.1	6.63, dd (7.7, 1.4)	119.1	6.75, d (7.7)	119.0	6.78, dd (7.9, 1.1)
5	142.5		142.3		142.8	
6	116.8	6.60, d (1.4)	115.0	6.77, s	116.0	6.75, d (1.1)
7	135.6		135.4		87.2	
8	130.1	5.38, td (1.3, 7.2)	130.8	5.43, td (1.1, 7.1)	39.5	2.14, 2.40, m
9	27.2	2.15, m	24.5	2.25, m	29.7	1.71, 1.86, m
10	40.0	1.33, m	44.3	1.60, m	86.4	3.65, m
11	28.9	1.61, m	71.4		34.4	1.75, m
12	23.0	0.93, d (6.6)	29.2	1.23, s	18.9	0.93, d (6.7)
13	23.0	0.93, d (6.6)	29.2	1.23, s	19.5	1.03, d (6.7)
14	17.3	1.94, s	17.2	1.96, s	29.5	1.50, s
15	36.1	3.80, s	65.0	4.5, s	64.9	4.50, s
16	129.2					
17	141.9					
18	145.9					
19	114.8	6.47, d (1.3)				
20	129.7					
21	122.9	6.34, d (1.3)				
22	20.9	2.13, s				

^a^*δ* in ppm; *J* in Hz; **3**, **4**, and **6** in methanol-*d*_4_.

**Table 3:** ^1^H and ^13^C NMR spectroscopic data (500/100 MHz) for **5**^a^.

No.	*δ*_C_	*δ*_H_	No.	*δ*_C_	*δ*_H_
1 (1′)	157.0		9 (9′)	23.0	1.27, m
2 (2′)	131.9		10 (10′)	40.5	1.13, m
3 (3′)	127.7	7.10, d (7.9)	11 (11′)	29.0	1.48, m
4 (4′)	119.8	6.78, d (7.9)	12 (12′)	23.0	0.82, d (6.6)
5 (5′)	139.5		13 (13′)	23.0	0.82, d (6.6)
6 (6′)	117.1	6.76, s	14 (14′)	29.0	1.58, s
7 (7′)	78.0		15 (15′)	72.6	4.44, s
8 (8′)	44.0	1.77, 1.88, m			

^a^*δ* in ppm, *J* in Hz, **5** in methanol-*d*_4_.

**Table 4:** α-Glucosidase inhibitory activities.

Compounds	1	2	3	4	5	6	7	8	9	10	11	Acarbose ^a^
IC_50_ (μM)	165.2	129.9	4.5	-	3.1	-	532.5	-	-	1.5	2.3	840.2

- means no activity; ^a^ positive control.

## Supplementary Materials

The following are available online at http://www.mdpi.com/1660-3397/16/9/307/s1, Figures S1–S44: The HRESIMS, NMR, and ECD spectra of compounds **1**–**6**.
