# Supplementary material for: α-Glucosidase Inhibitors: Diphenyl Ethers and Phenolic Bisabolane Sesquiterpenoids from the Mangrove Endophytic Fungus Aspergillus flavus QQSG-3
Source: Mar Drugs. 2018 Sep 1;16(9):307. doi: 10.3390/md16090307 (PMC6165285; doi:10.3390/md16090307)
Supplement: Supplementary file 1 [file marinedrugs-16-00307-s001.pdf]

Supporting Information

α-Glucosidase Inhibitors: Diphenyl Ethers and Phenolic Bisabolane Sesquiterpenoids from Mangrove Endophytic Fungus *Aspergillus flavus* QQSG-3

Yingnan Wu[1], Yan Chen[2], Xishan Huang[1], Yahong Pan[1], Zhaoming Liu[1, 3,*], Tao Yan[4], Wenhao Cao[4] and Zhigang She[1, 2,*]

[1] School of Chemistry, Sun Yat-Sen University, Guangzhou 510275, China; wuyn3@mail2.sysu.edu.cn (Y.W.); huangxsh9@mail.sysu.edu.cn (X.H); pan16a@126.com (Y.P.)

[2] School of Marine Sciences, Sun Yat-Sen University, South China Sea Bio-Resource Exploitation and Utilization Collaborative Innovation Center, Guangzhou 510006, China; chenyan27@mail2.sysu.edu.cn (Y.C.)

[3] State Key Laboratory of Applied Microbiology, Southern China, Guangzhou 510075, China

[4] CAS Key Laboratory of Tropical Marine Bio-resources and Ecology, Guangdong Key Laboratory of Marine Materia, RNAM Center for Marine Microbiology, South China Sea Institute of Oceanology, Chinese Academy of Sciences, Guangzhou 510301, China; yantao@scsio.ac.cn (T.Y.); chromo@163.com (W.C.)

* Correspondence: cesshzhg@mail.sysu.edu.cn (Z.S.); liuzhaom@mail2.sysu.edu.cn (Z.L.); Tel.: +86-20-8411-3356 (Z.S.)

CONTENT

Fig.S1. HRESIMS spectrum of compound 1 4

Fig.S2. ^{1}H NMR (500 MHz, $CDCl_3$) spectrum of compound 1 4

Fig.S3.^{13}C NMR (125 MHz, $CDCl_3$) spectrum of 1 5

Fig.S4. ^{1}H-^{1}H COSY ($CDCl_3$) spectrum of compound 1 5

Fig.S5. HSQC ($CDCl_3$)spectrum of compound 1 6

Fig.S6. HMBC ($CDCl_3$) spectrum of compound 1 6

Fig.S7. NOESY ($CDCl_3$) spectrum of compound 1 7

Fig.S8. HRESIMS spectrum of compound 2 7

Fig.S9. ^{1}H NMR (500 MHz, Methanol-d_4) spectrum of compound 2 8

Fig.S10. ^{13}C NMR (125 MHz, Methanol-d_4) spectrum of compound 2 8

Fig.S11. ^{1}H-^{1}H COSY (Methanol-d_4) spectrum of compound 2 9

Fig.S12. HSQC (Methanol-d_4) spectrum of compound 2 9

Fig.S13. HMBC (Methanol-d_4) spectrum of compound 2 10

Fig.S14. NOESY (Methanol-d_4) spectrum of compound 2 10

Fig.S15. HRESIMS spectrum of compound 3 11

Fig.S16. ^{1}H NMR (500 MHz, Methanol-d_4) spectrum of compound 3 11

Fig.S17. ^{13}C NMR (125 MHz, Methanol-d_4) spectrum of compound 3 12

Fig.S18. ^{1}H-^{1}H COSY (Methanol-d_4) spectrum of compound 3 12

Fig.S19. HSQC (Methanol-d_4) spectrum of compound 3 13

Fig.S20. HMBC (Methanol-d_4) spectrum of compound 3 13

Fig.S21. NOEDIFF (Methanol-d_4， original) spectrum of compound 3 14

Fig.S22. NOEDIFF (Methanol-d_4， zoom in) spectrum of compound 3 14

Fig.S23. HRESIMS spectrum of compound 4 15

Fig.S24. ^{1}H NMR (500 MHz, Methanol-d_4) spectrum of compound 4 15

Fig.S25. ^{13}C NMR (125 MHz, Methanol-d_4) spectrum of compound 4 16

Fig.S26. ^{1}H-^{1}H COSY (Methanol-d_4) spectrum of compound 4 16

Fig.S27. HSQC (Methanol-d_4) spectrum of compound 4 17

Fig.S28. HMBC (Methanol-d_4) spectrum of compound 4 17

Fig.S29. NOEDIFF (Methanol-d_4) spectrum of compound 4 18

Fig.S30. HRESIMS spectrum of compound 5 18

Fig.S31. ^{1}H NMR (500 MHz, Methanol-d_4) spectrum of compound 5 19
Fig.S32. ^{13}C NMR (125 MHz, Methanol-d_4) spectrum of compound 5 19
Fig.S33. ^{1}H-^{1}H COSY (Methanol-d_4) spectrum of compound 5 20
Fig.S34. HSQC (Methanol-d_4) spectrum of compound 5 20
Fig.S35. HMBC (Methanol-d_4) spectrum of compound 5 21
Fig.S36. Experimental ECD spectrum of compound 5 21
Fig.S37. HRESIMS spectrum of compound 6 22
Fig.S38. ^{1}H NMR (500 MHz, Methanol-d_4) spectrum of compound 6 22
Fig.S39. ^{13}C NMR (125 MHz, Methanol-d_4) spectrum of compound 6 23
Fig.S40. ^{1}H- ^{1}H COSY (Methanol-d_4) spectrum of compound 6 23
Fig.S41. HSQC (Methanol-d_4) spectrum of compound 6 24
Fig.S42. HMBC (Methanol-d_4) spectrum of compound 6 24
Fig.S43. NOESY (Methanol-d_4) spectrum of compound 6 25
Fig.S44. Experimental ECD spectrum of compound 6 25

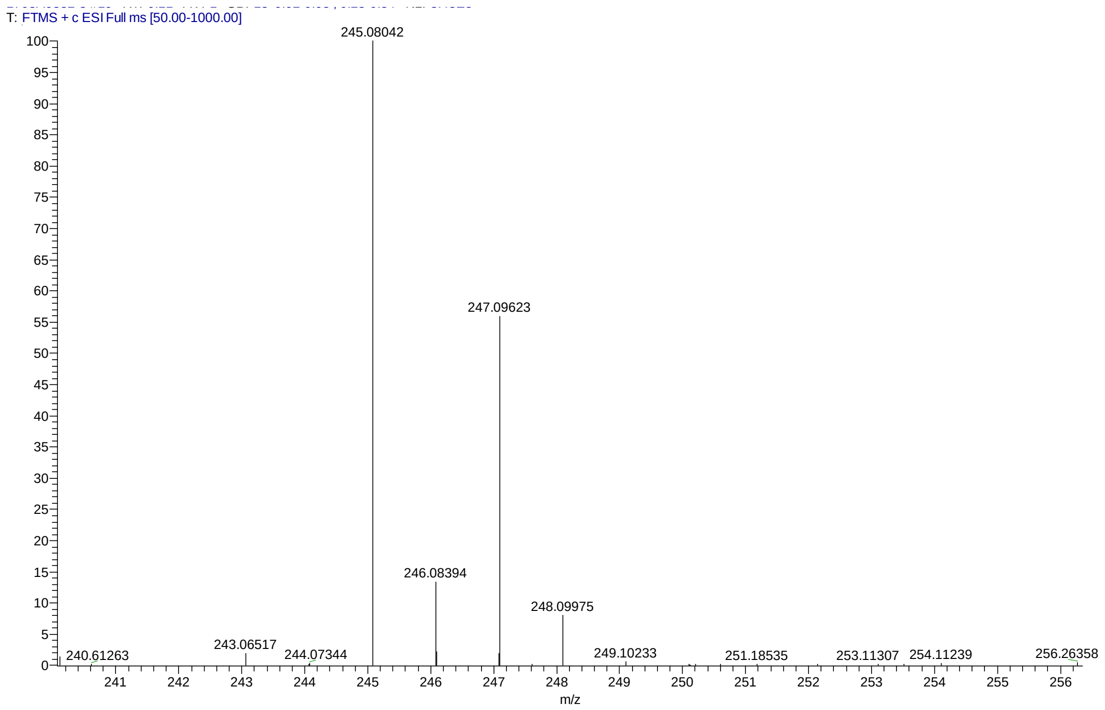

Fig.S1. HRESIMS spectrum of compound **1**

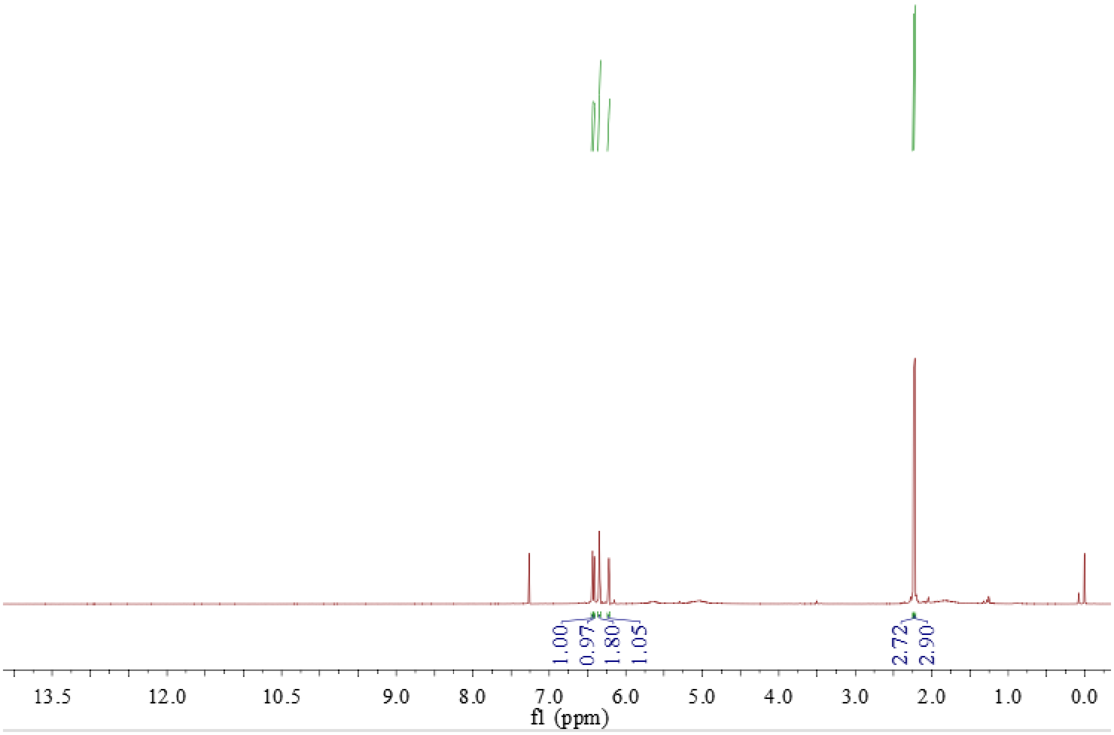

Fig.S2. ^{1}H NMR (500 MHz, $CDCl_3$) spectrum of compound **1**

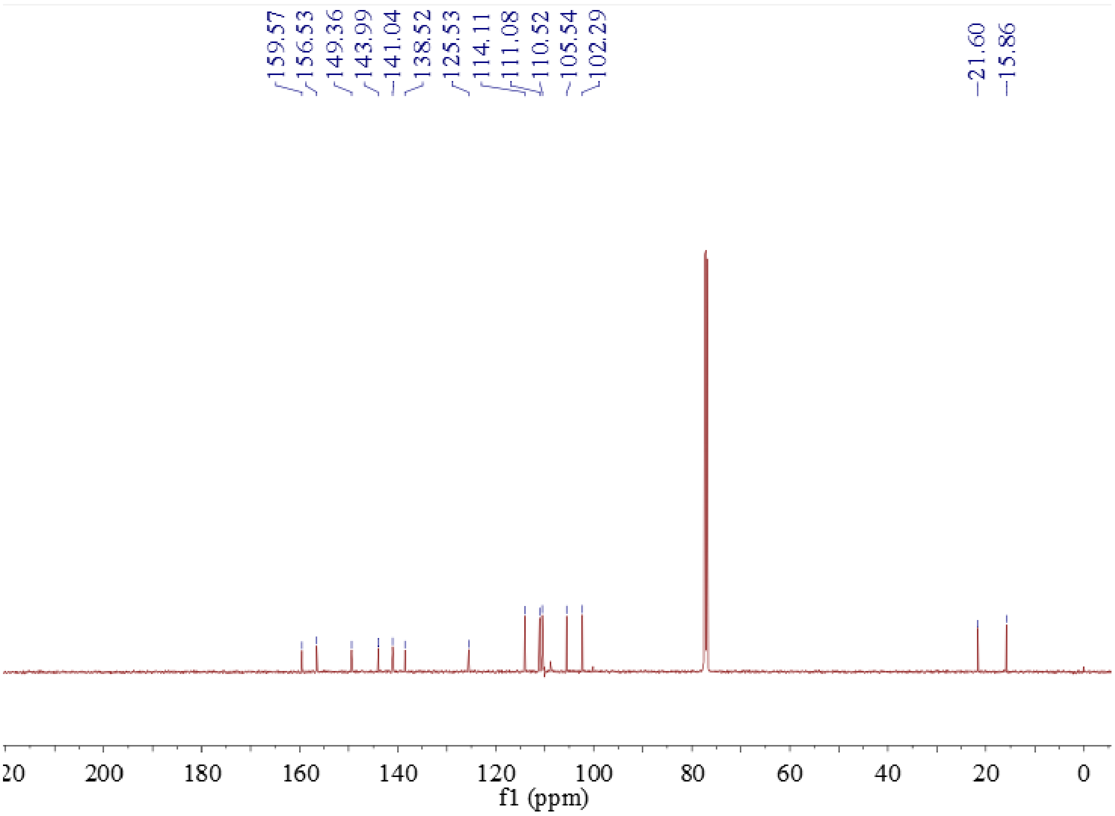

Fig.S3. ^{13}C NMR (125 MHz, $CDCl_3$) spectrum of **1**

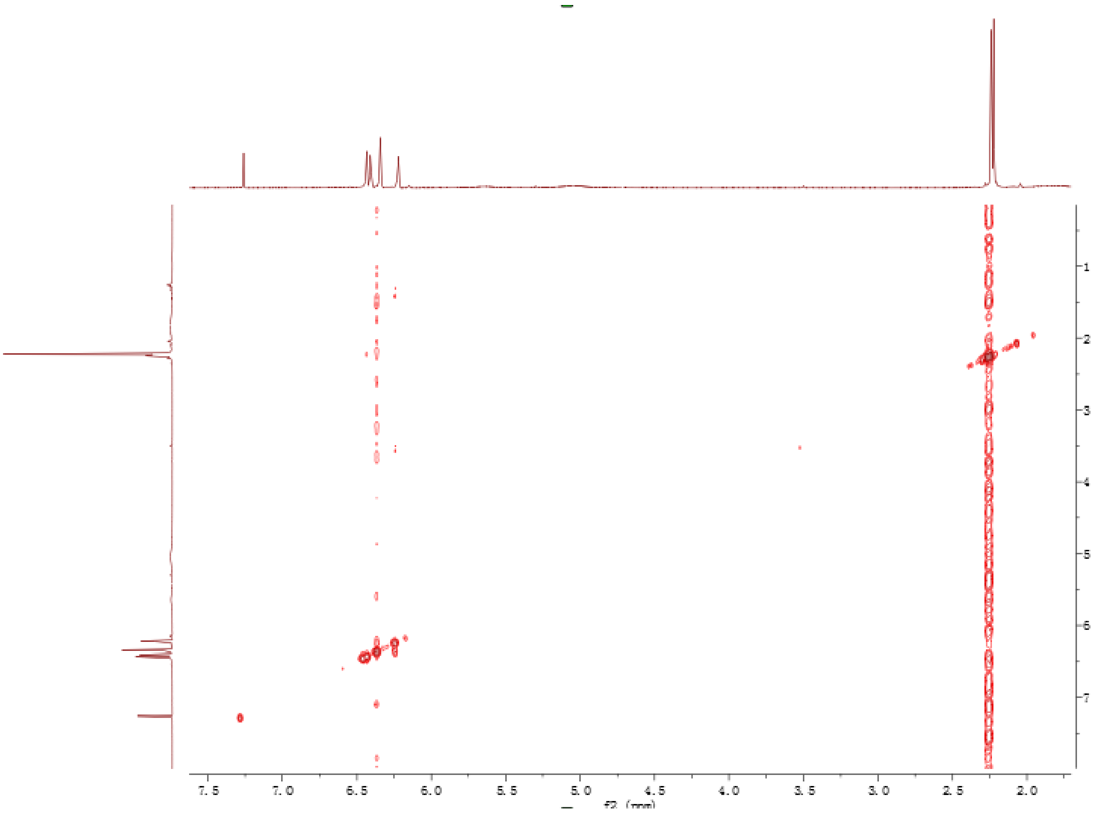

Fig.S4. ^{1}H-^{1}H COSY ($CDCl_3$) spectrum of compound **1**

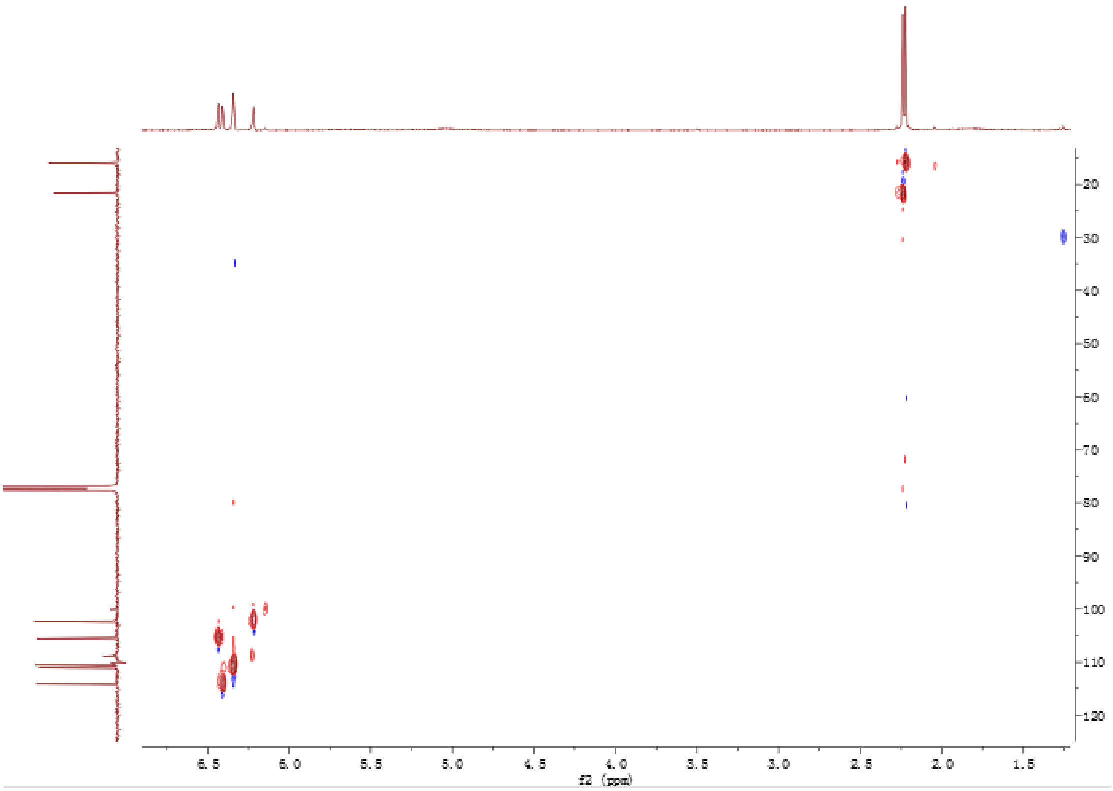

Fig.S5. HSQC ($CDCl_3$)spectrum of compound **1**

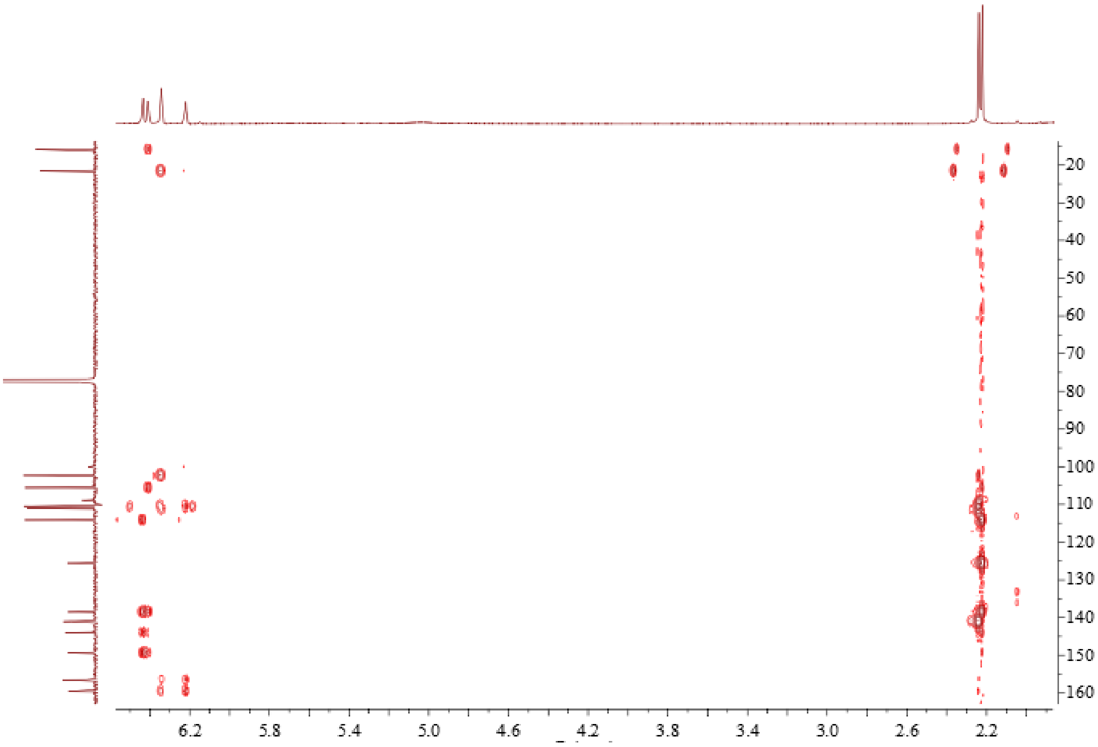

Fig.S6. HMBC ($CDCl_3$) spectrum of compound **1**

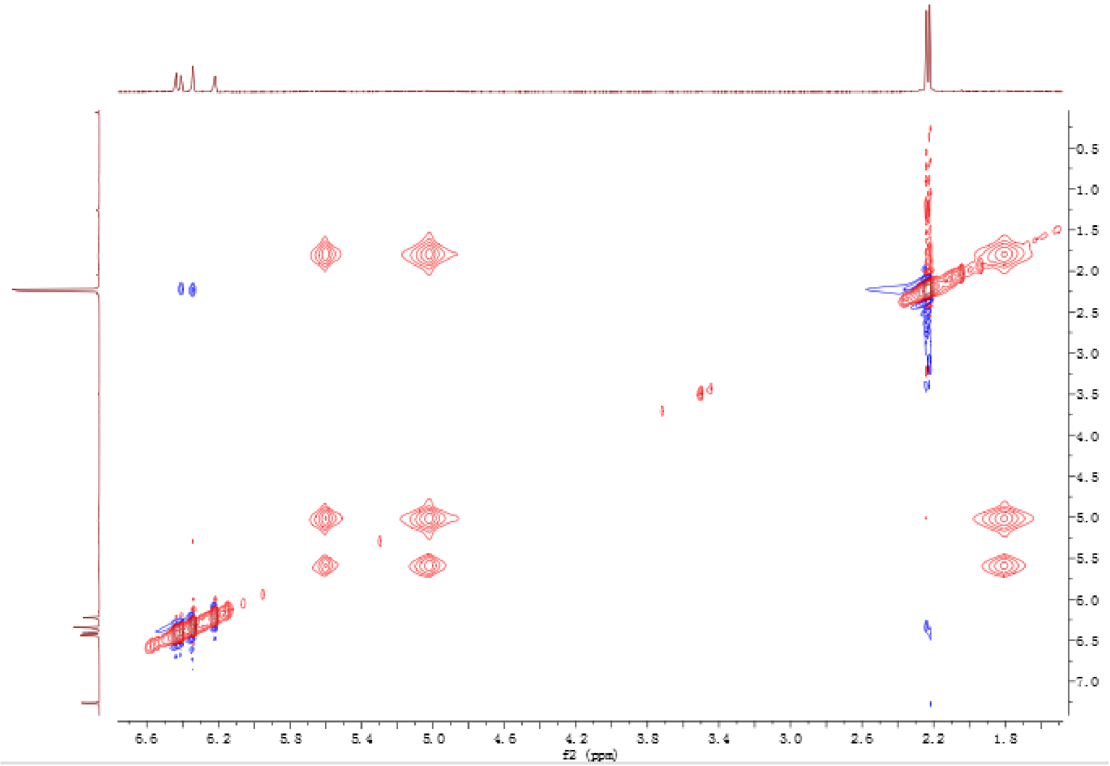

Fig.S7. NOESY ($CDCl_3$) spectrum of compound **1**

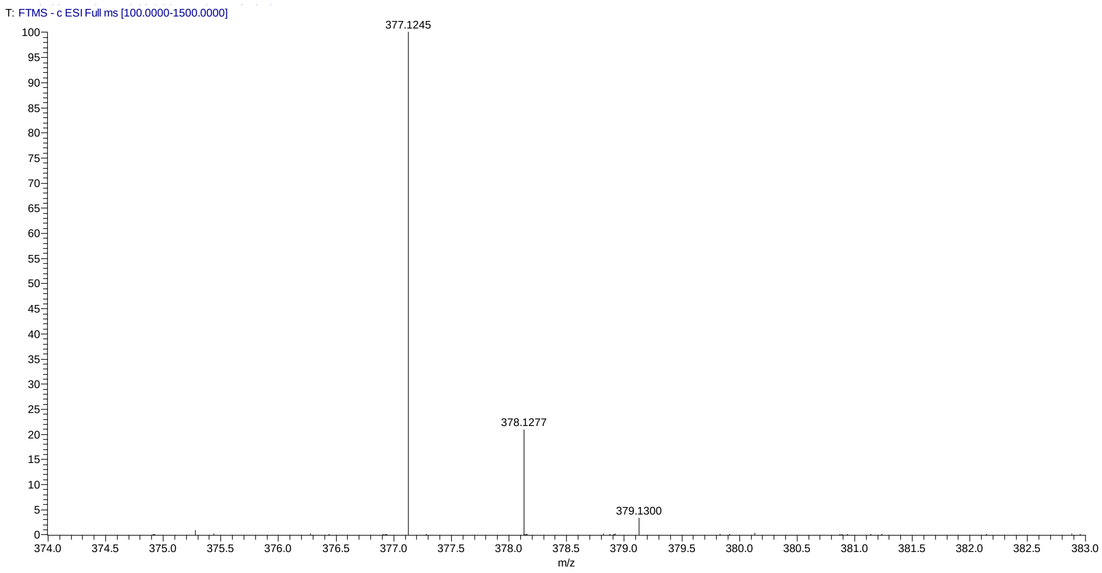

Fig.S8. HRESIMS spectrum of compound **2**

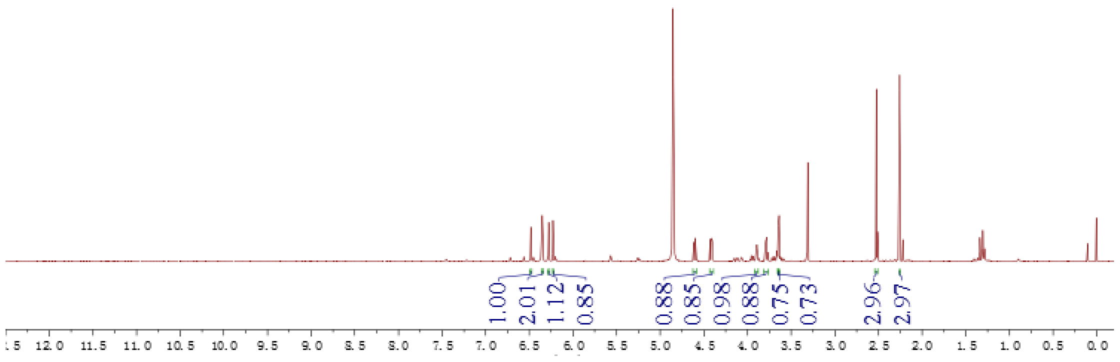

Fig.S9. ^{1}H NMR (500 MHz, Methanol-d_4) spectrum of compound **2**

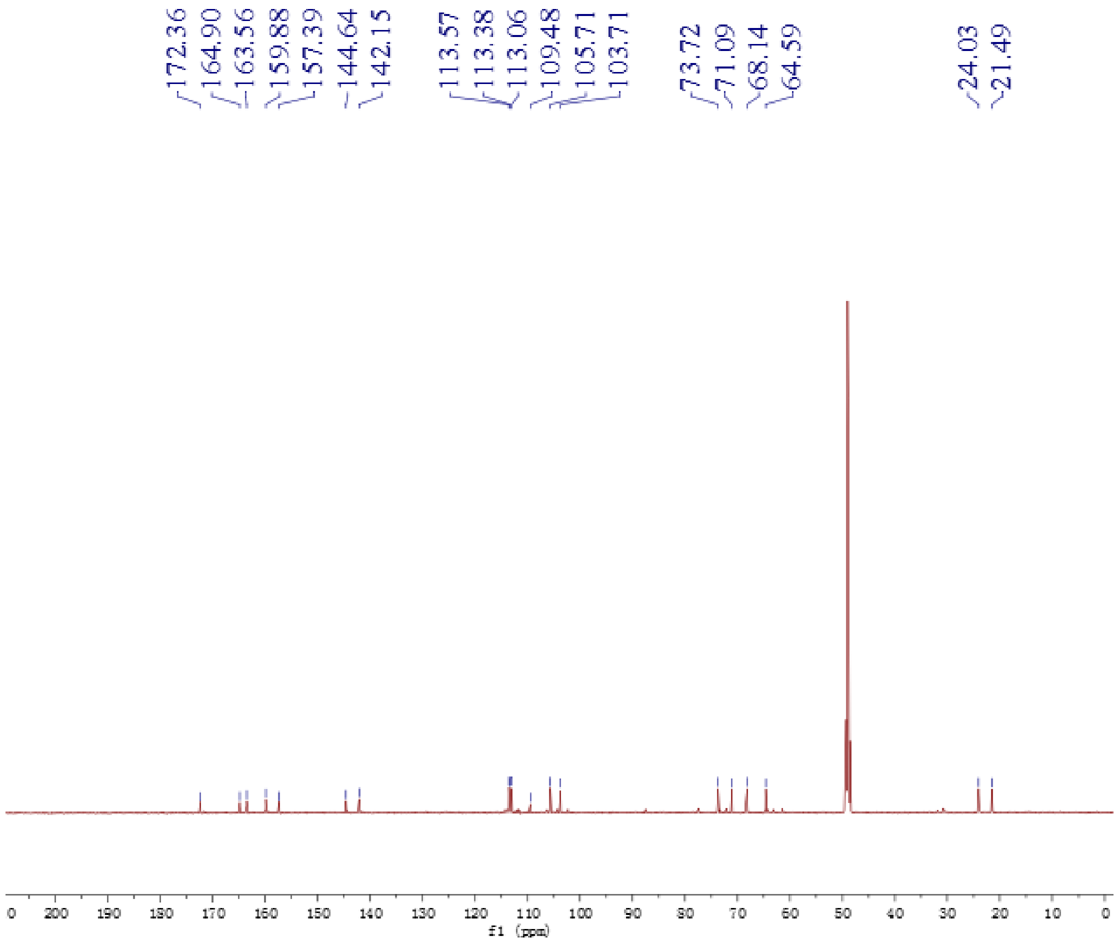

Fig.S10. ^{13}C NMR (125 MHz, Methanol-d_4) spectrum of compound **2**

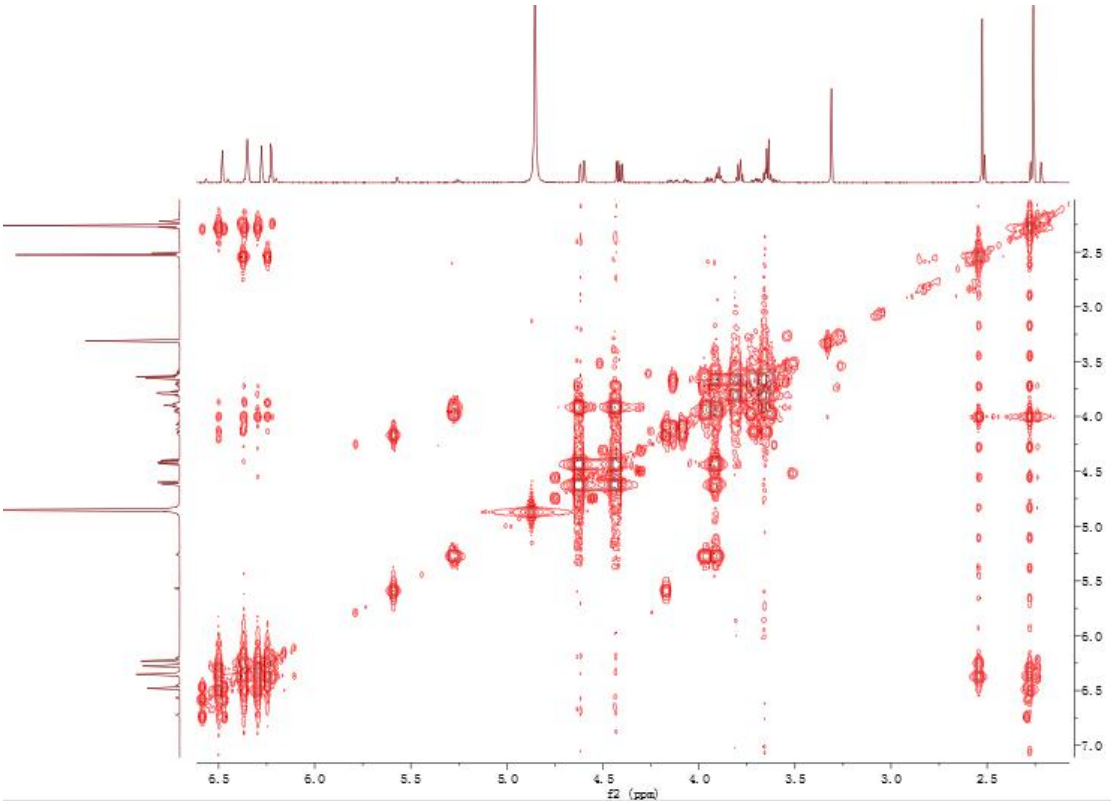

Fig.S11. ^{1}H-^{1}H COSY (Methanol-d_4) spectrum of compound **2**

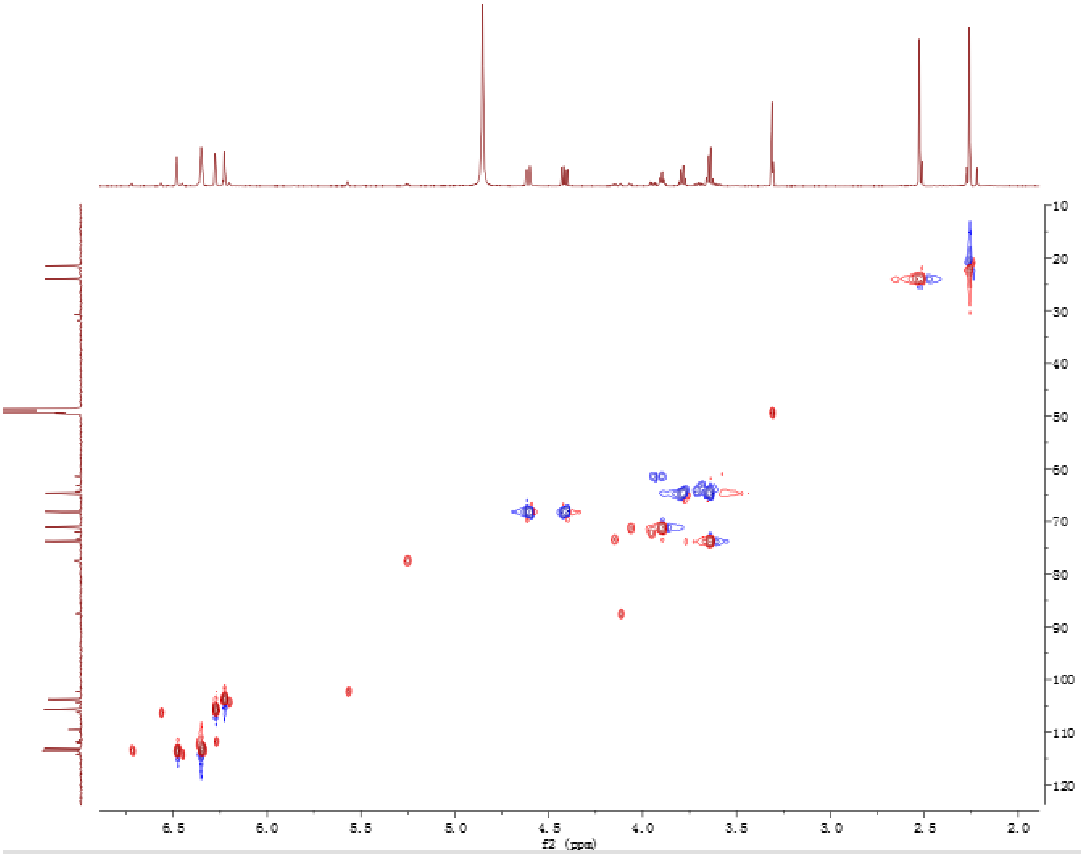

Fig.S12. HSQC (Methanol-d_4) spectrum of compound **2**

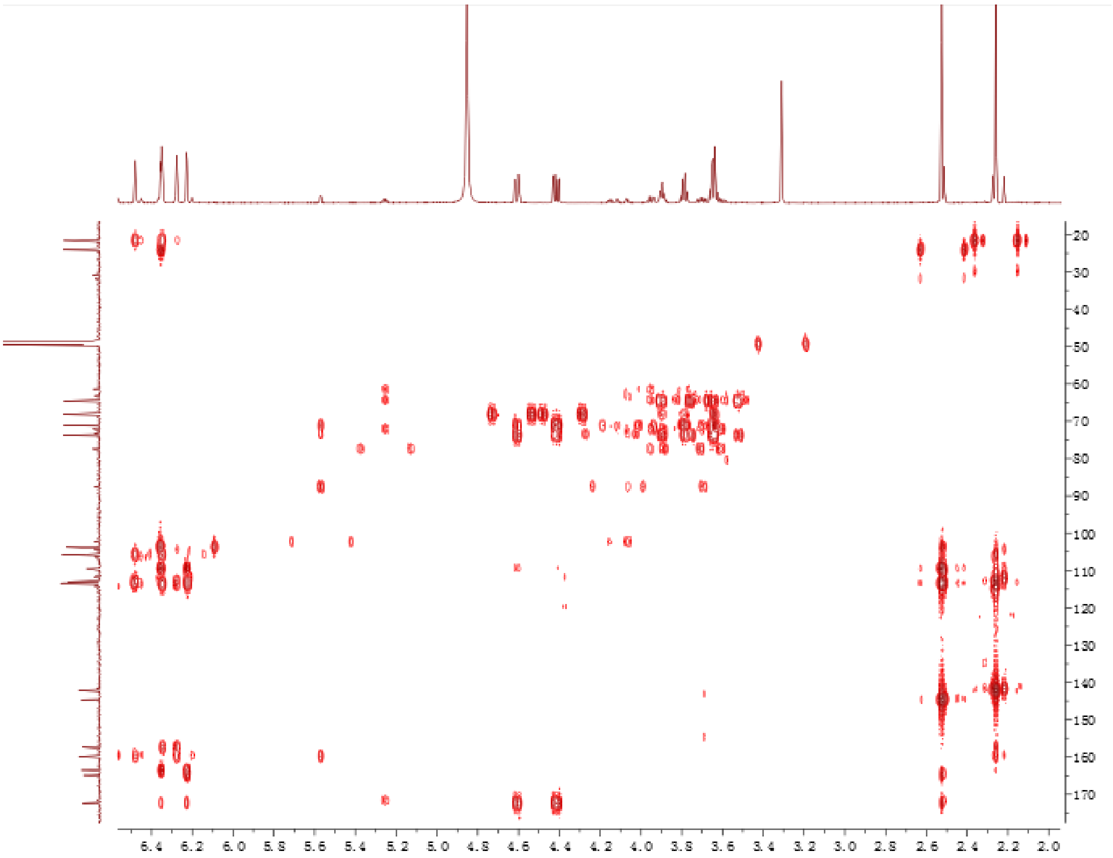

Fig.S13. HMBC (Methanol-d_4) spectrum of compound **2**

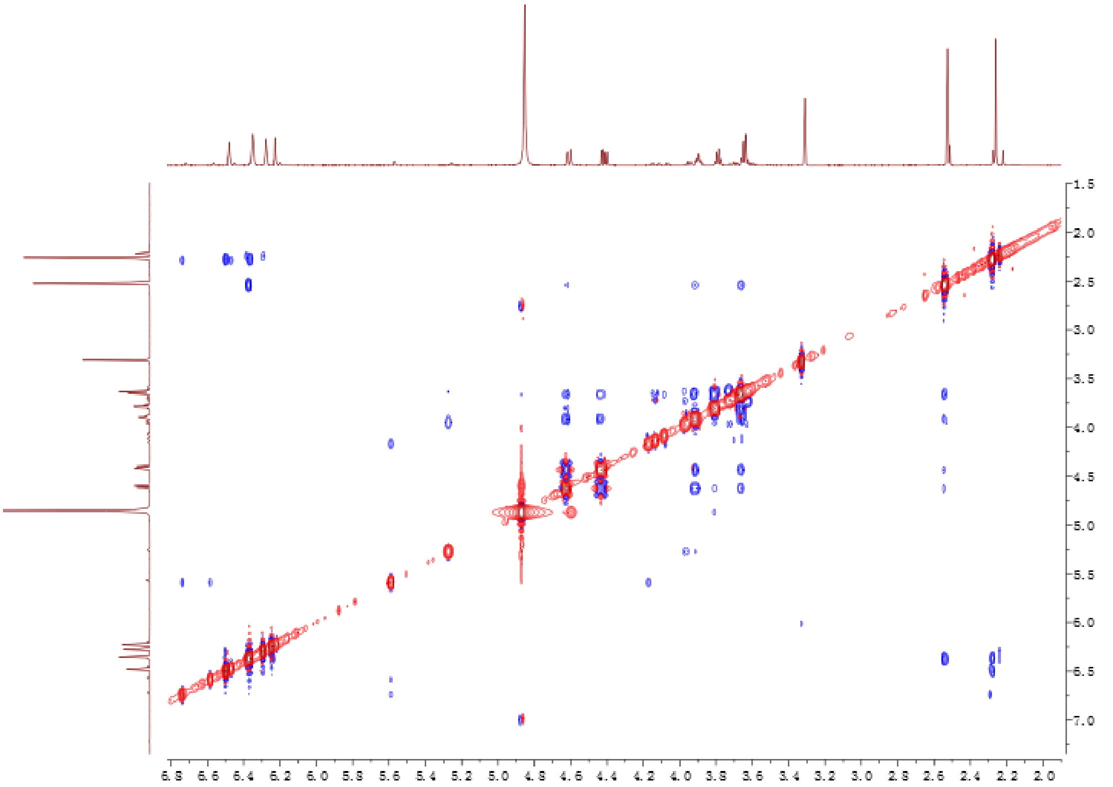

Fig.S14. NOESY (Methanol-d_4) spectrum of compound **2**

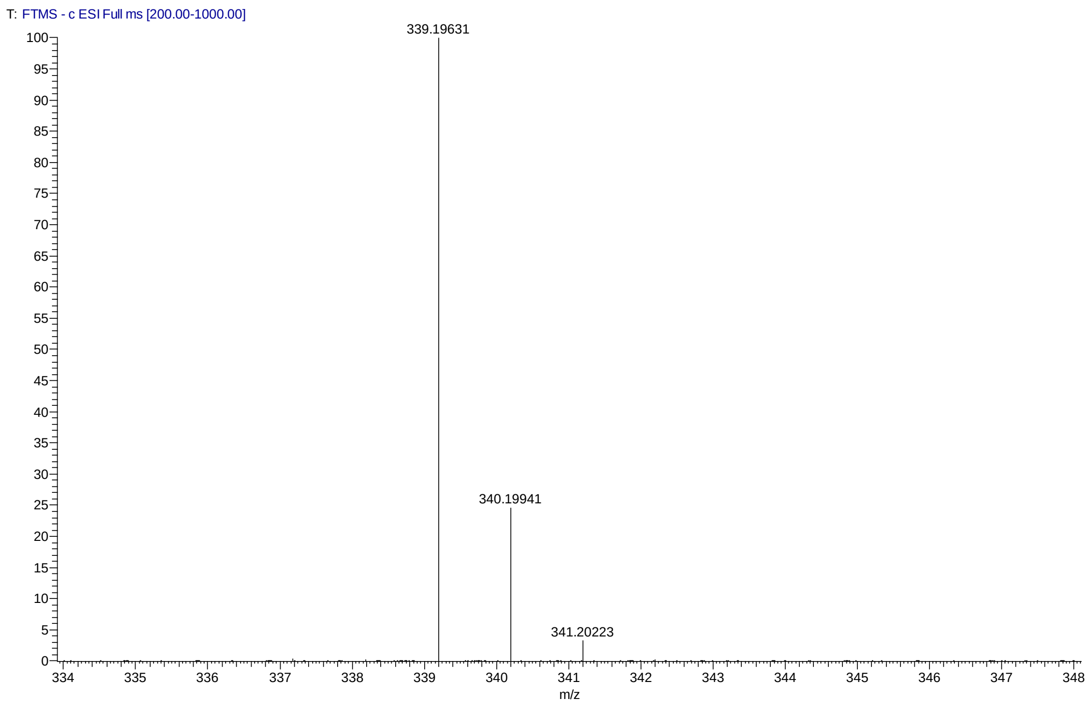

Fig.S15. HRESIMS spectrum of compound **3**

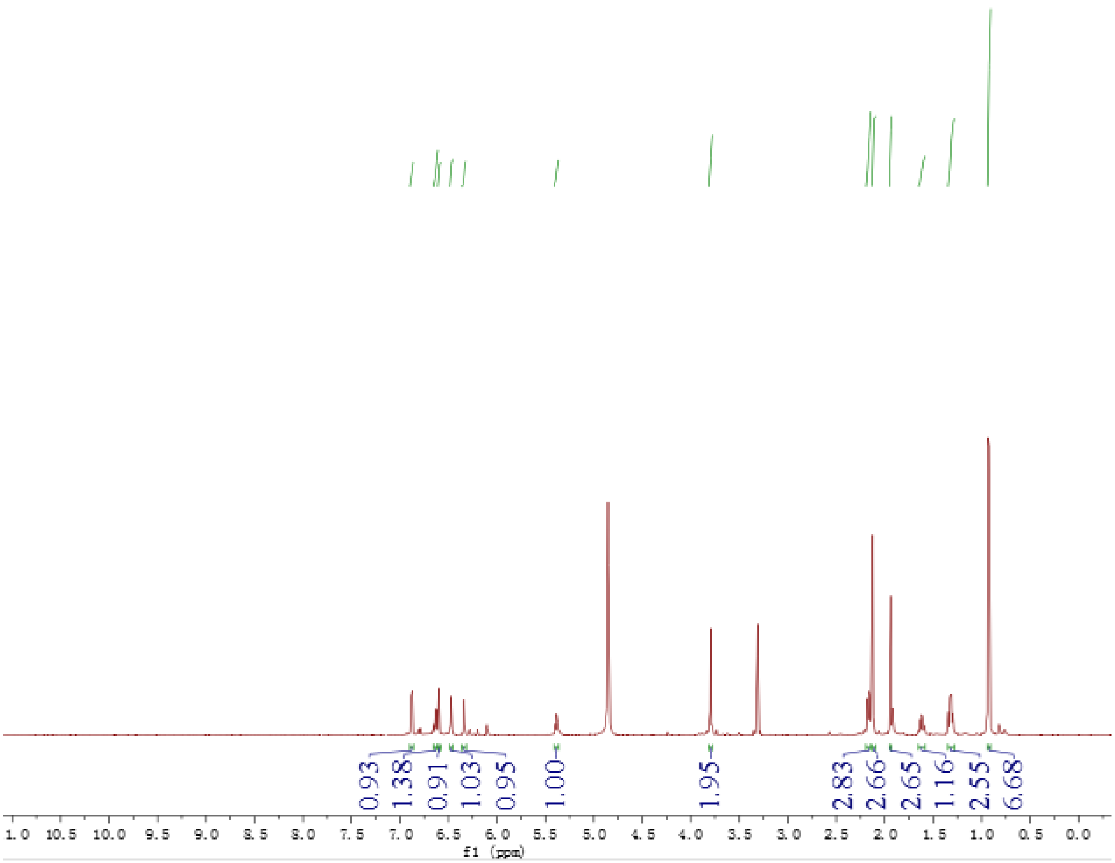

Fig.S16. ^{1}H NMR (500 MHz, Methanol-d_4) spectrum of compound **3**

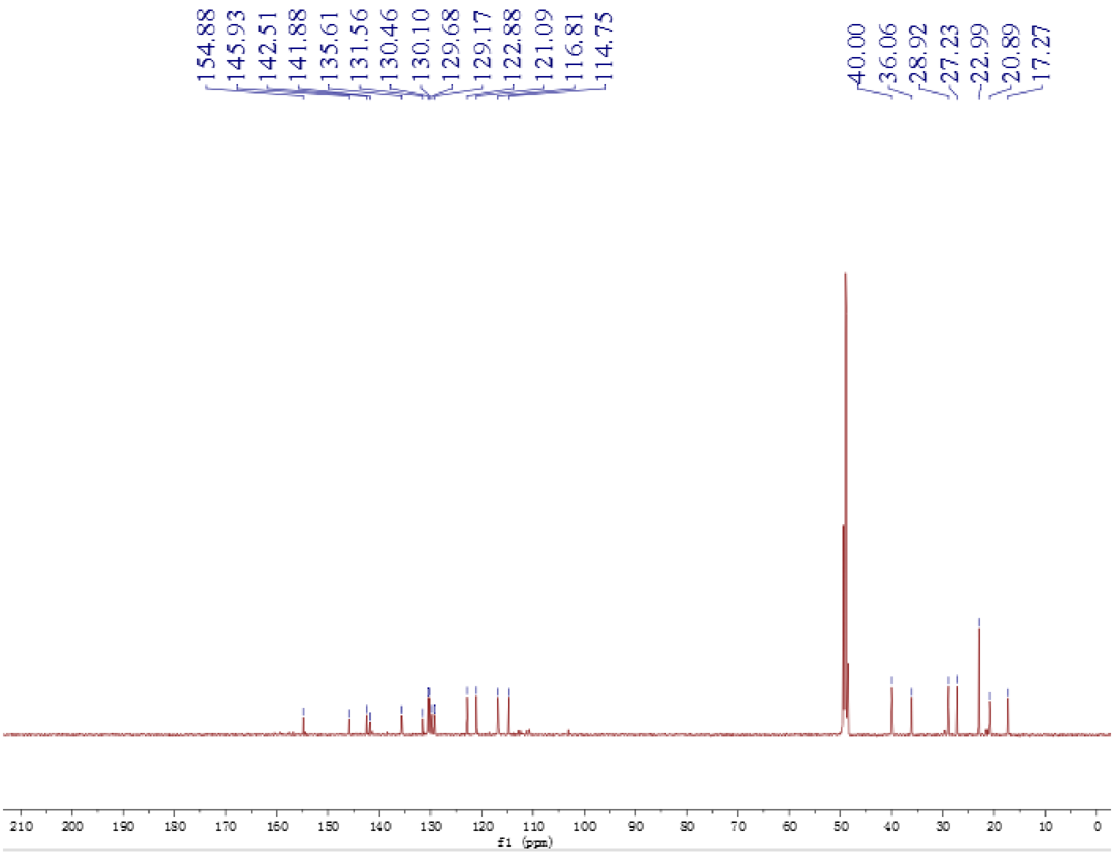

Fig.S17. ^{13}C NMR (125 MHz, Methanol-d_4) spectrum of compound **3**

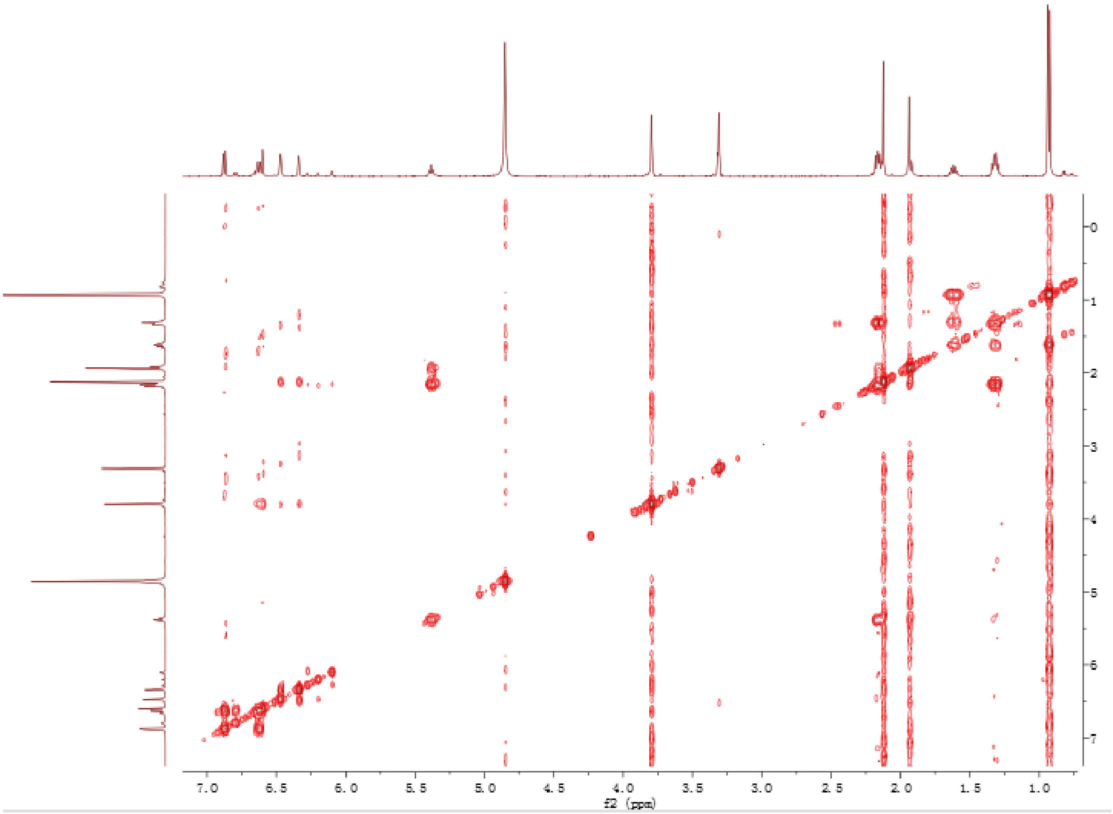

Fig.S18. ^{1}H-^{1}H COSY (Methanol-d_4) spectrum of compound **3**

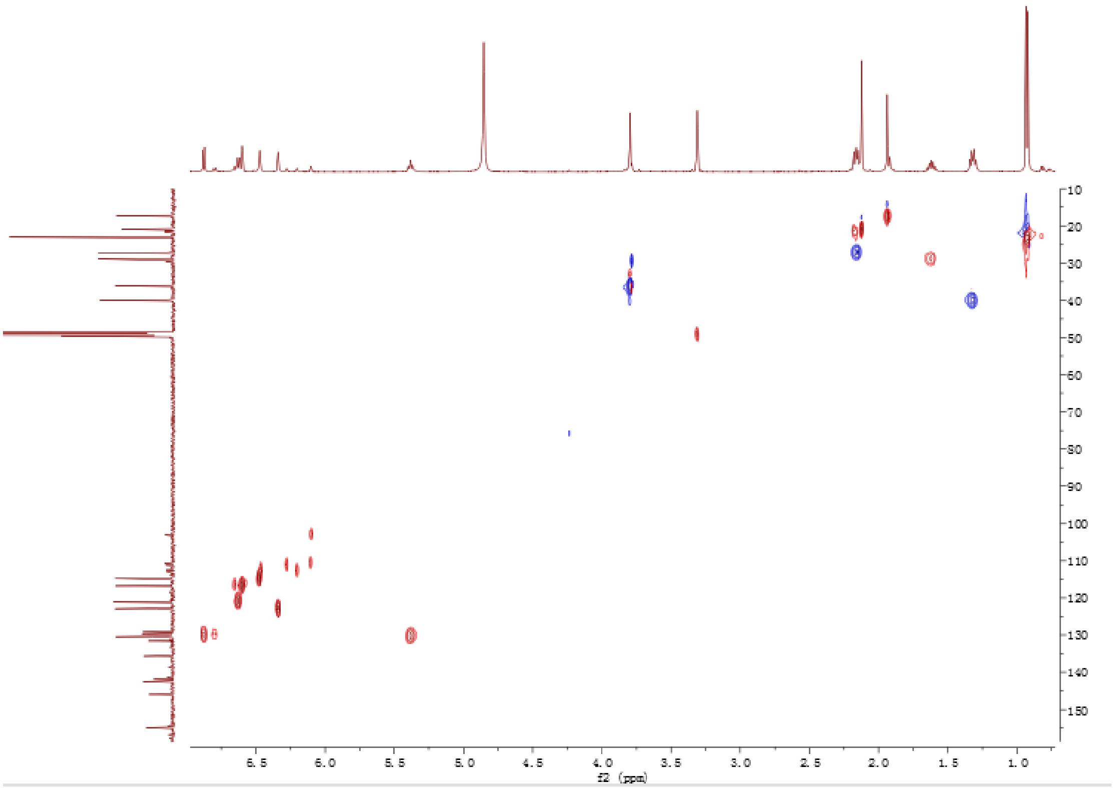

Fig.S19. HSQC (Methanol-d_4) spectrum of compound **3**

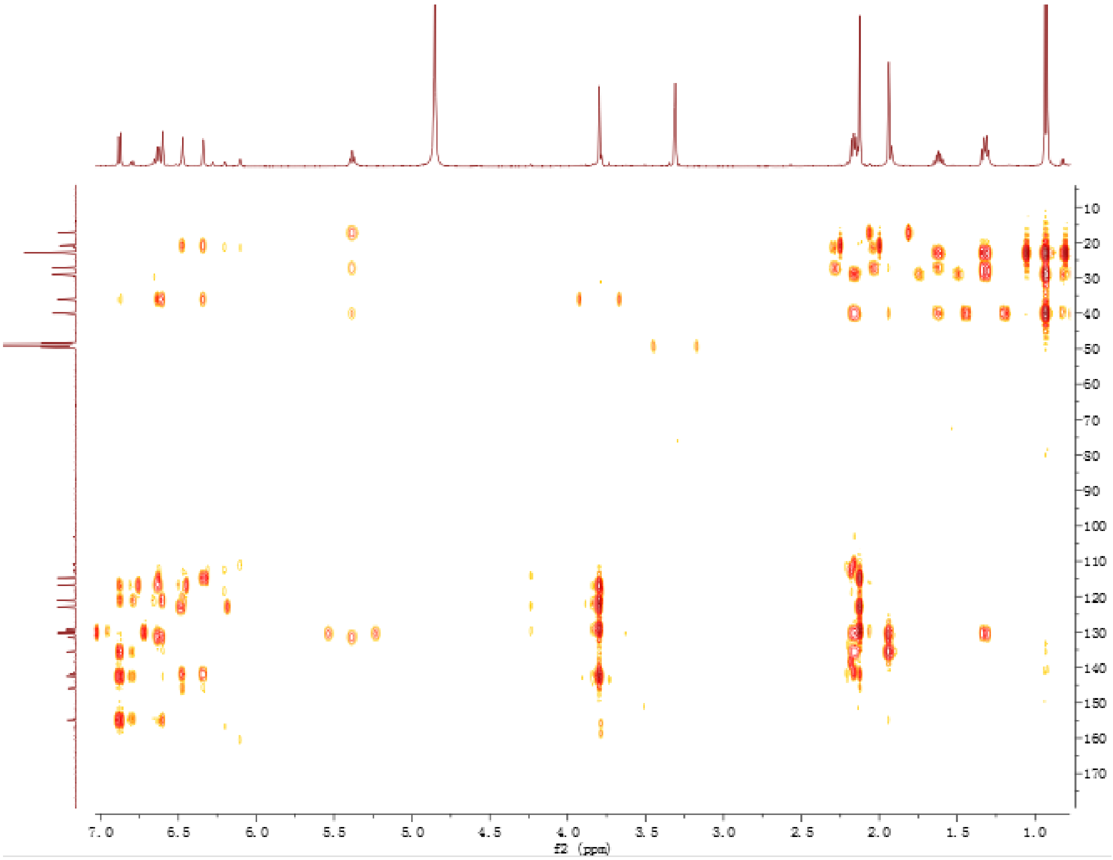

Fig.S20. HMBC (Methanol-d_4) spectrum of compound **3**

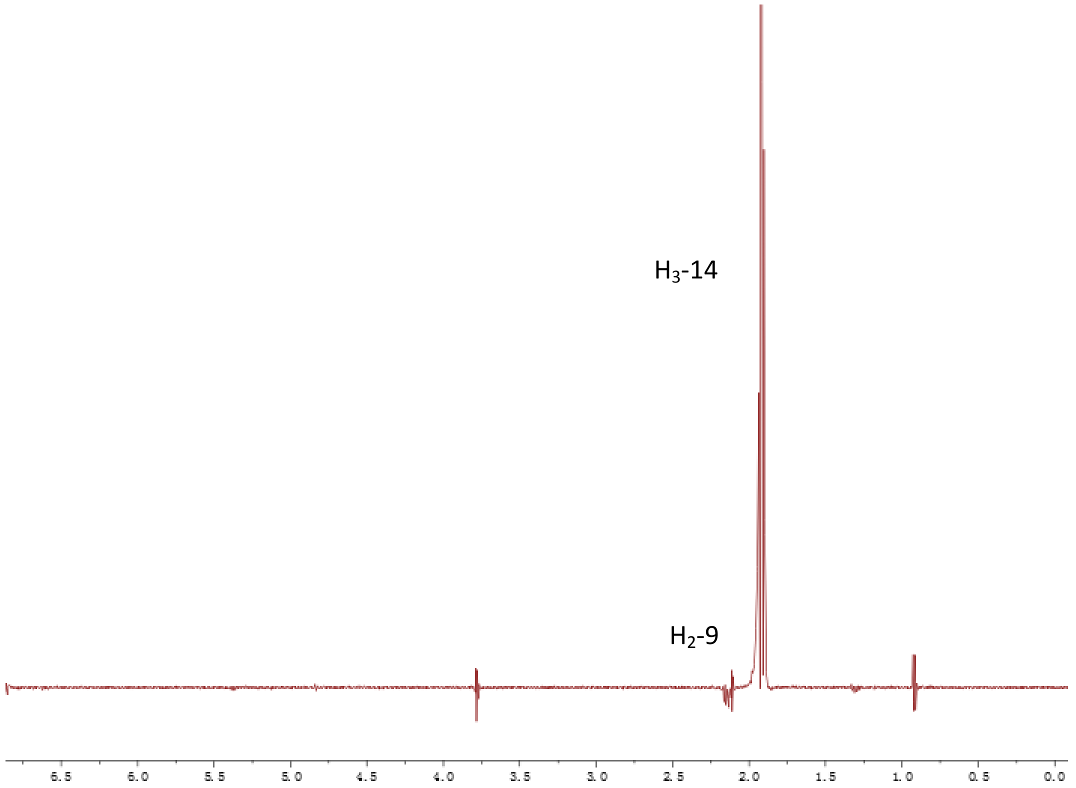

Fig.S21. NOEDIFF (Methanol-d_4， original) spectrum of compound **3**

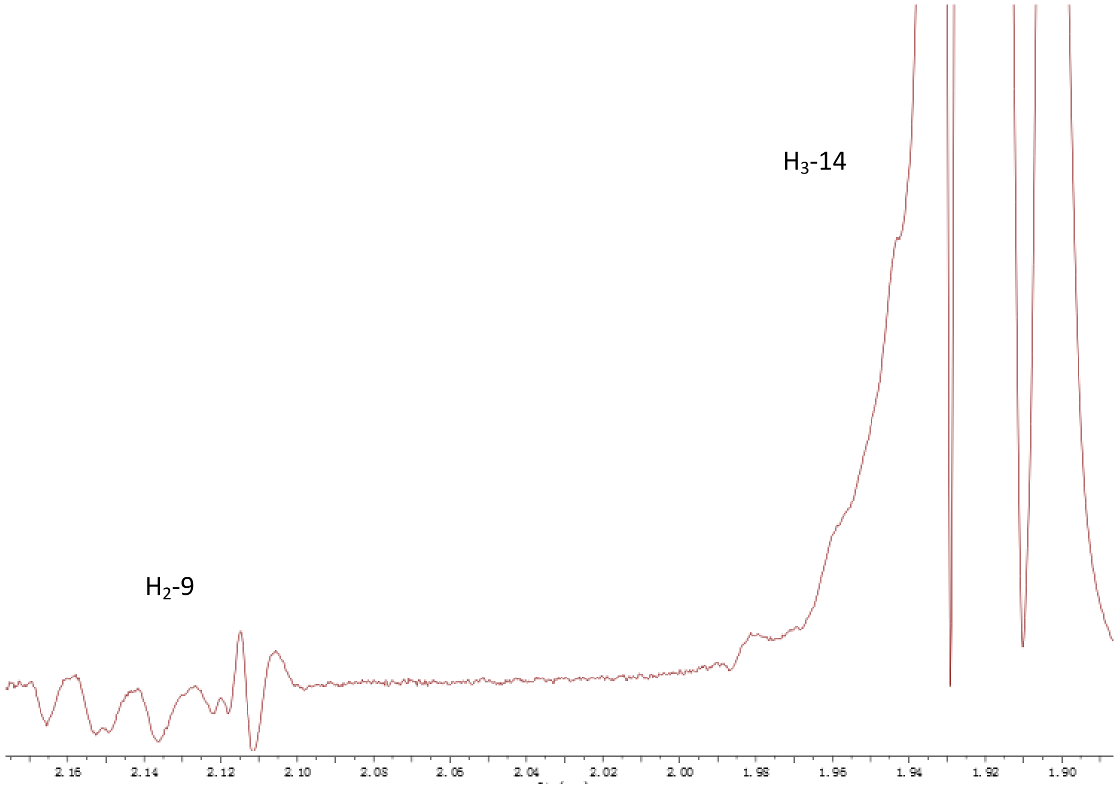

Fig.S22. NOEDIFF (Methanol-d_4， zoom in) spectrum of compound **3**

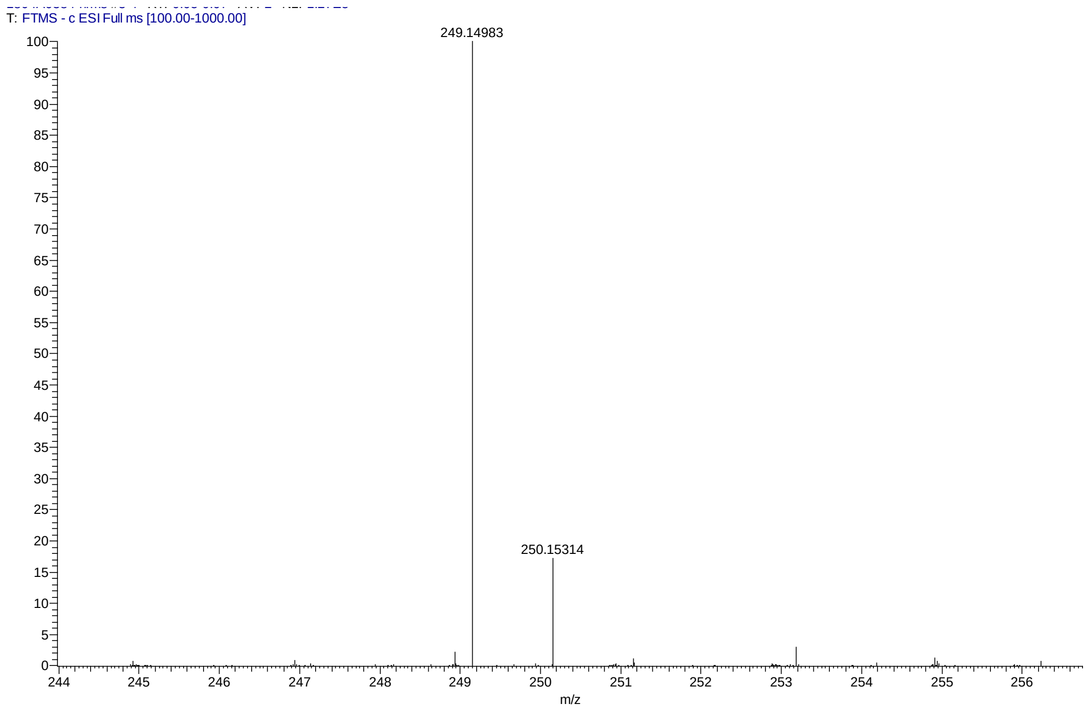

Fig.S23. HRESIMS spectrum of compound **4**

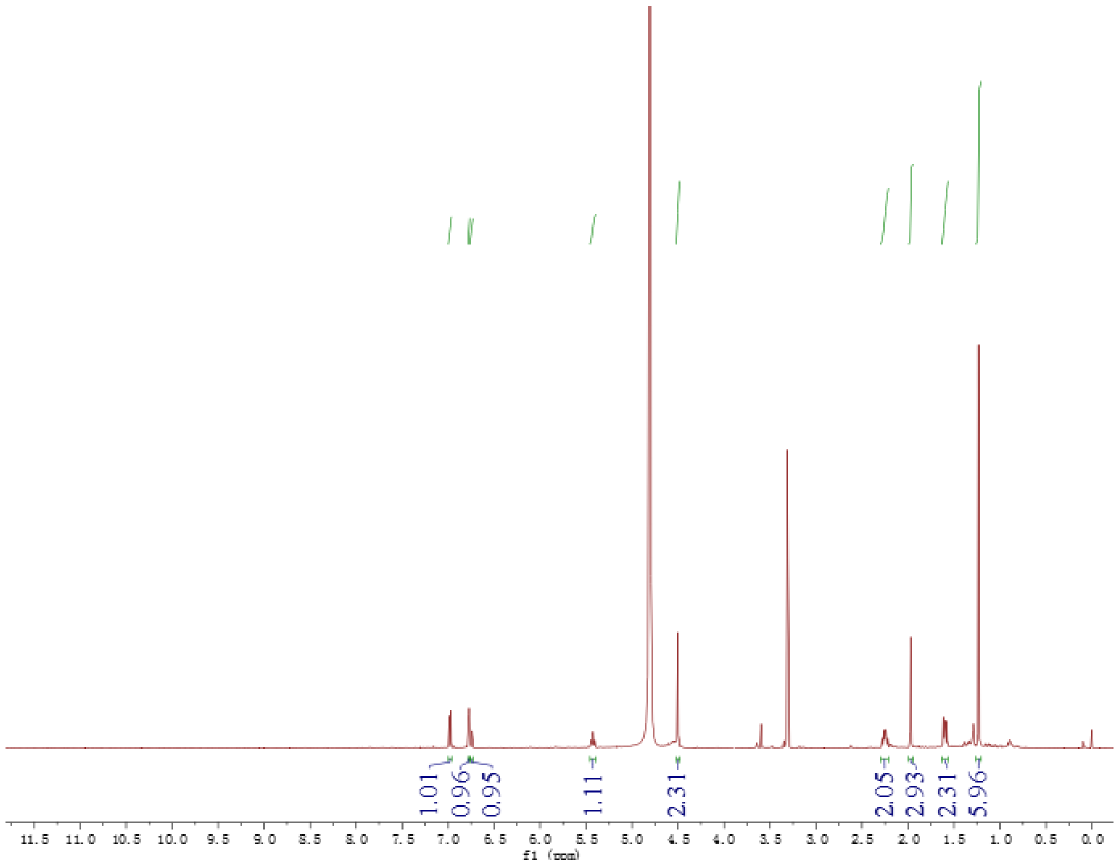

Fig.S24. ^{1}H NMR (500 MHz, Methanol-d_4) spectrum of compound **4**

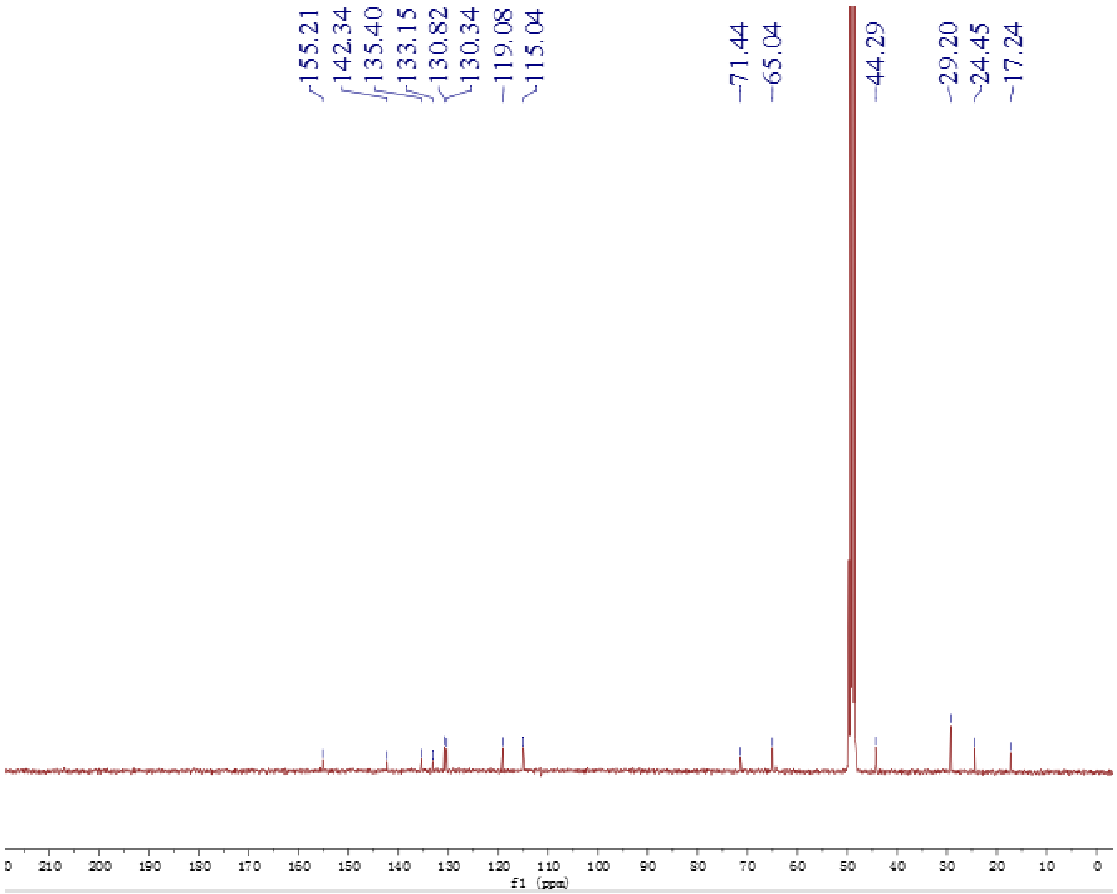

Fig.S25. ^{13}C NMR (125 MHz, Methanol-d_4) spectrum of compound **4**

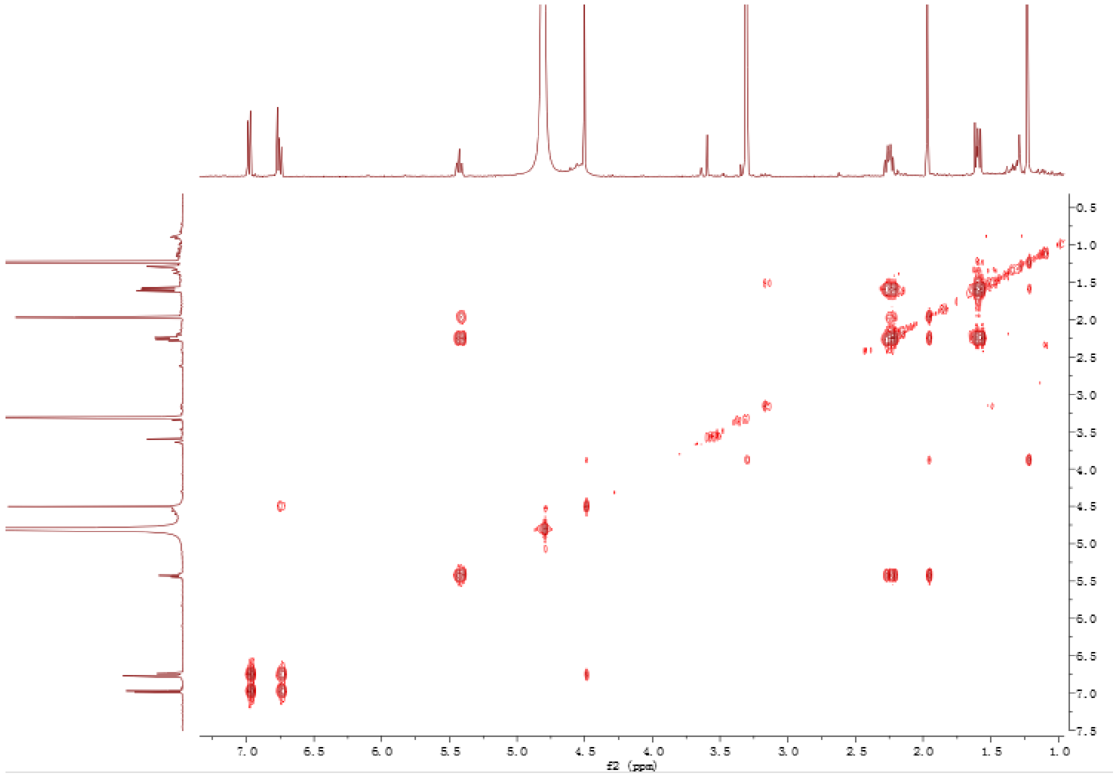

Fig.S26. ^{1}H-^{1}H COSY (Methanol-d_4) spectrum of compound **4**

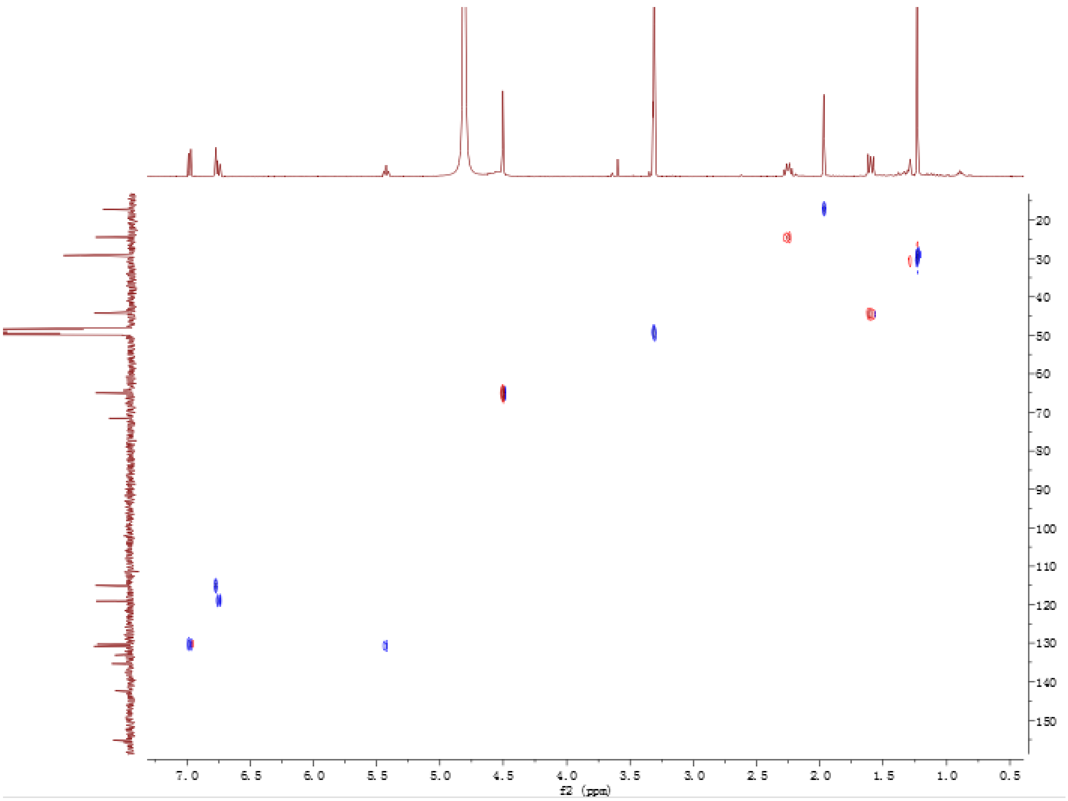

Fig.S27. HSQC (Methanol-d_4) spectrum of compound **4**

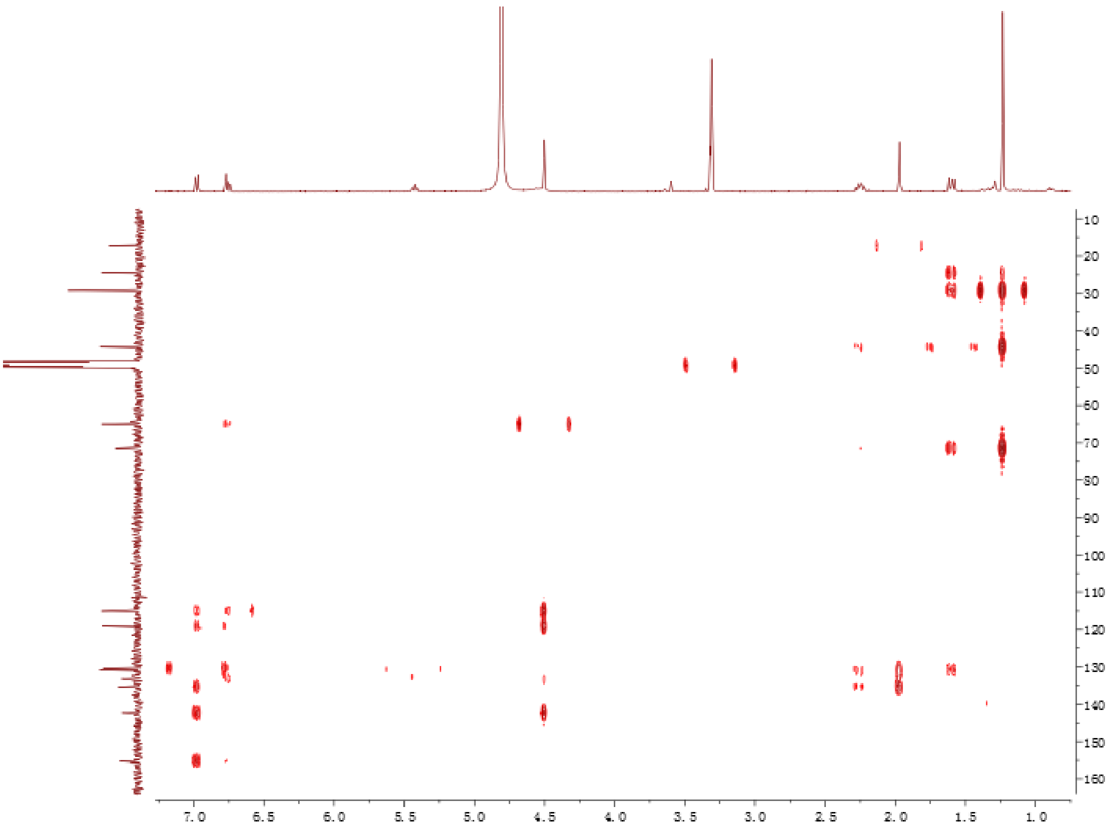

Fig.S28. HMBC (Methanol-d_4) spectrum of compound **4**

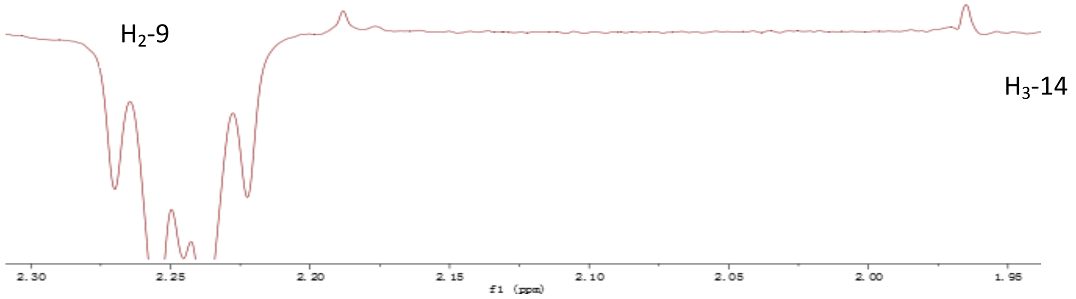

Fig.S29. NOEDIFF (Methanol-d_4) spectrum of compound **4**

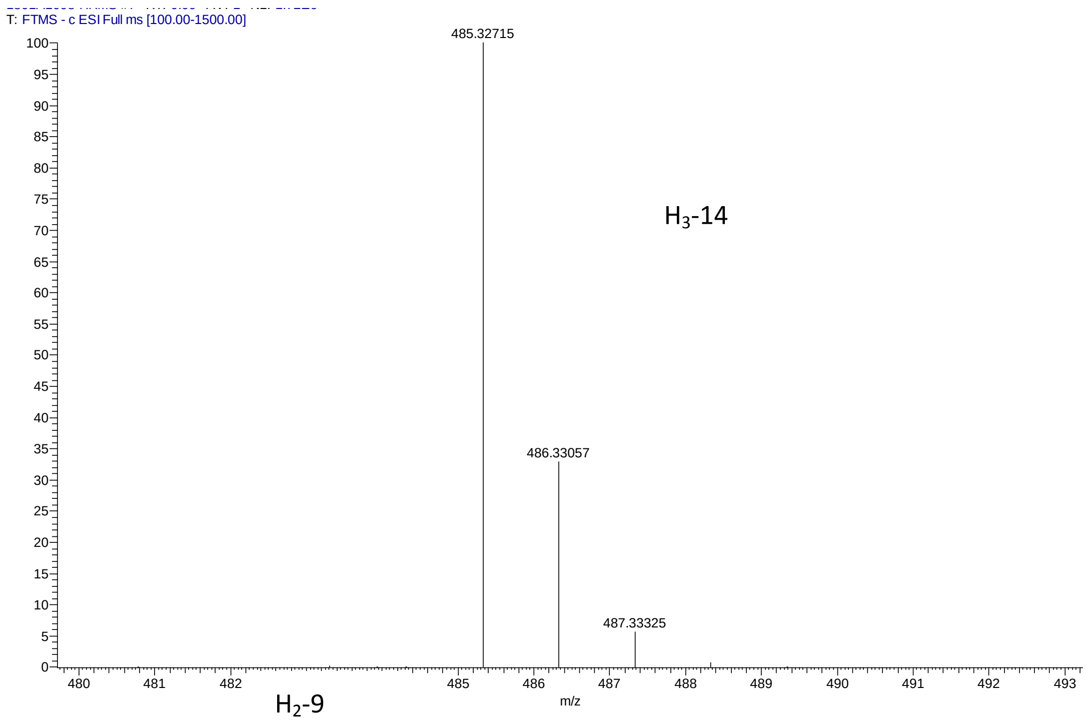

Fig.S30. HRESIMS spectrum of compound **5**

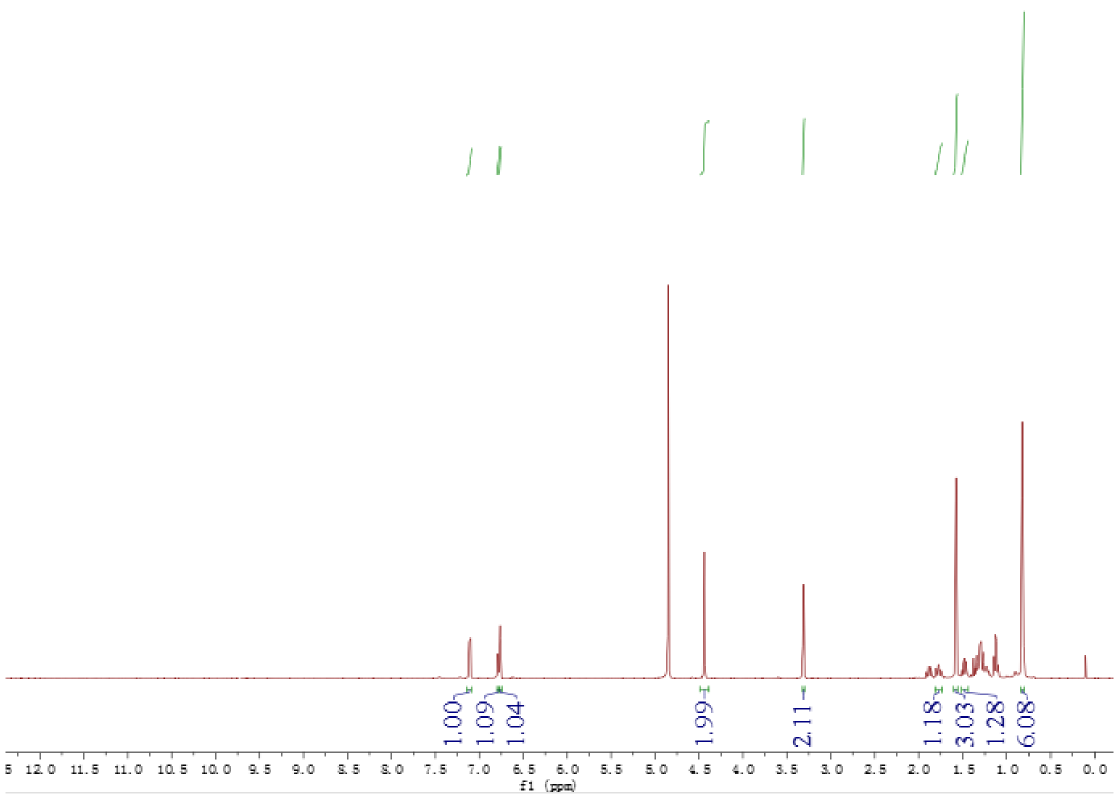

Fig.S31. ^{1}H NMR (500 MHz, Methanol-d_4) spectrum of compound **5**

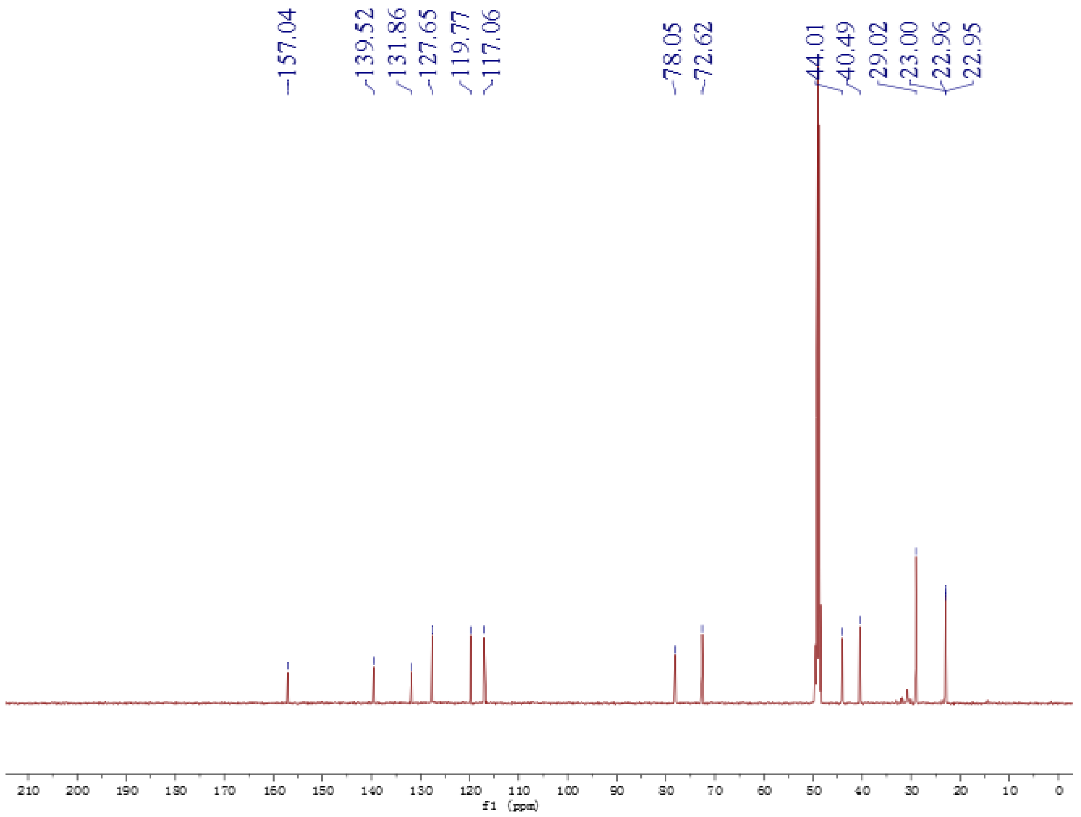

Fig.S32. ^{13}C NMR (125 MHz, Methanol-d_4) spectrum of compound **5**

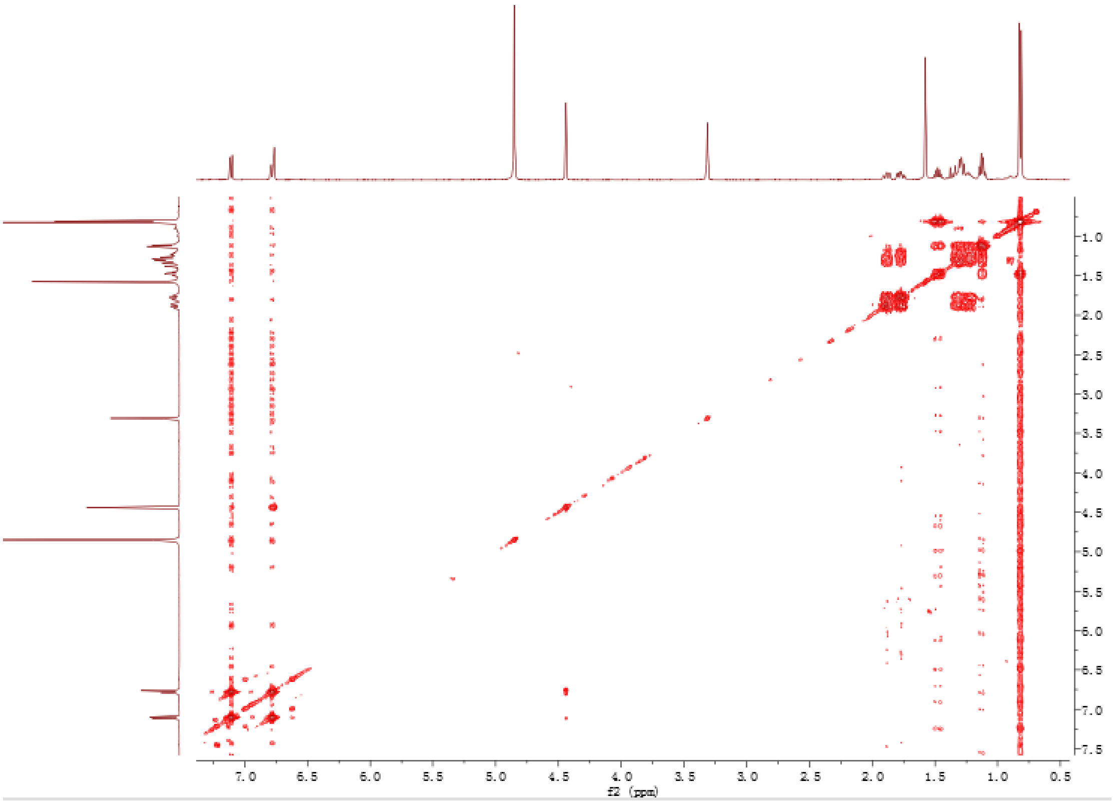

Fig.S33. ^{1}H-^{1}H COSY (Methanol-d_4) spectrum of compound **5**

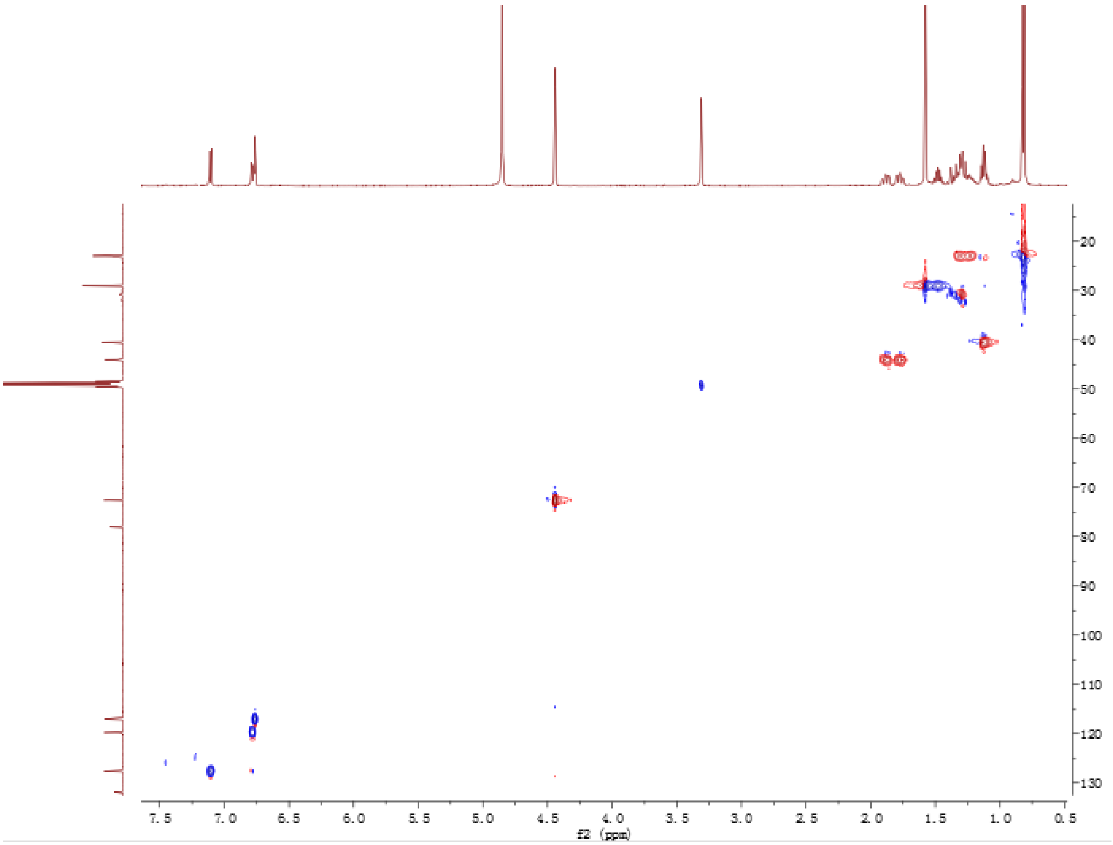

Fig.S34. HSQC (Methanol-d_4) spectrum of compound **5**

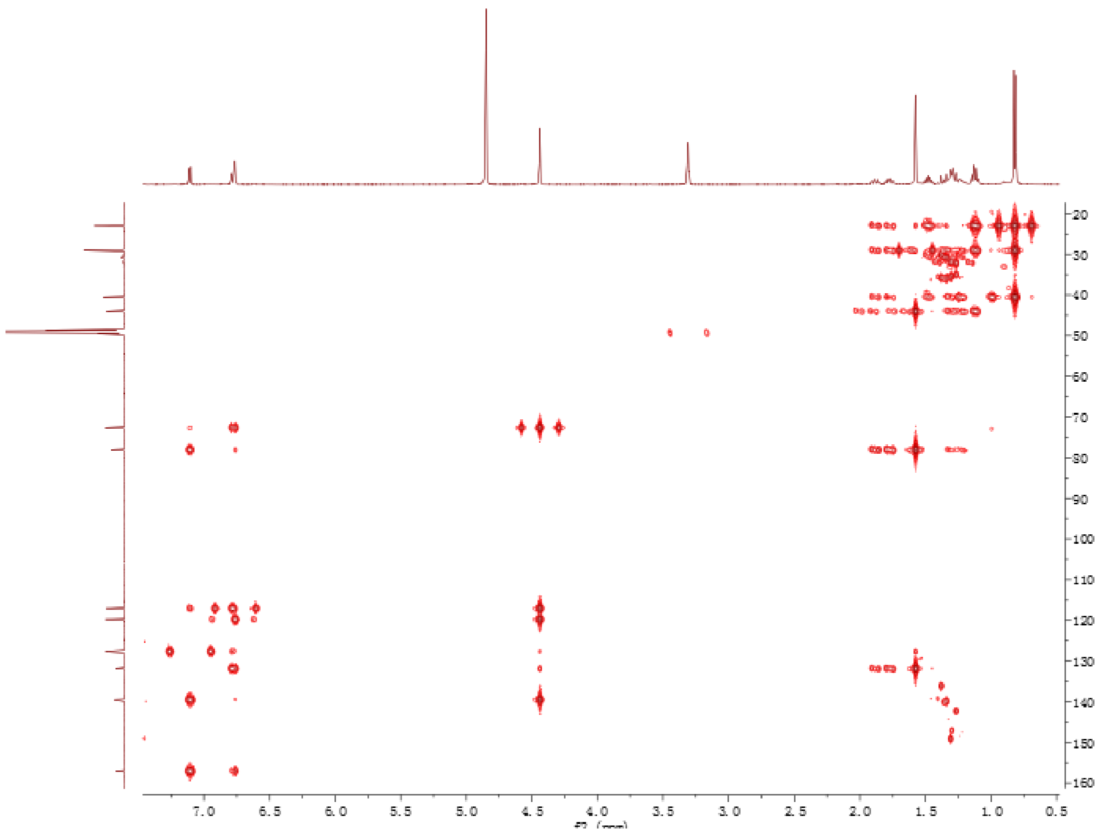

Fig.S35. HMBC (Methanol-d_4) spectrum of compound **5**

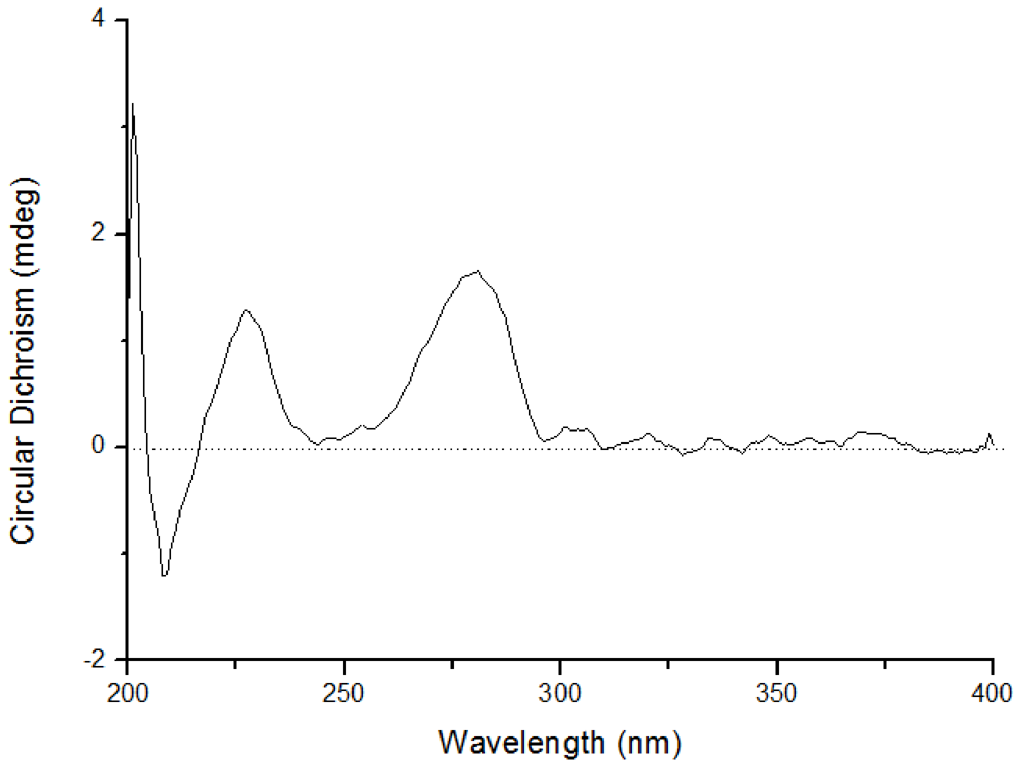

Fig.S36. Experimental ECD spectrum of compound **5**

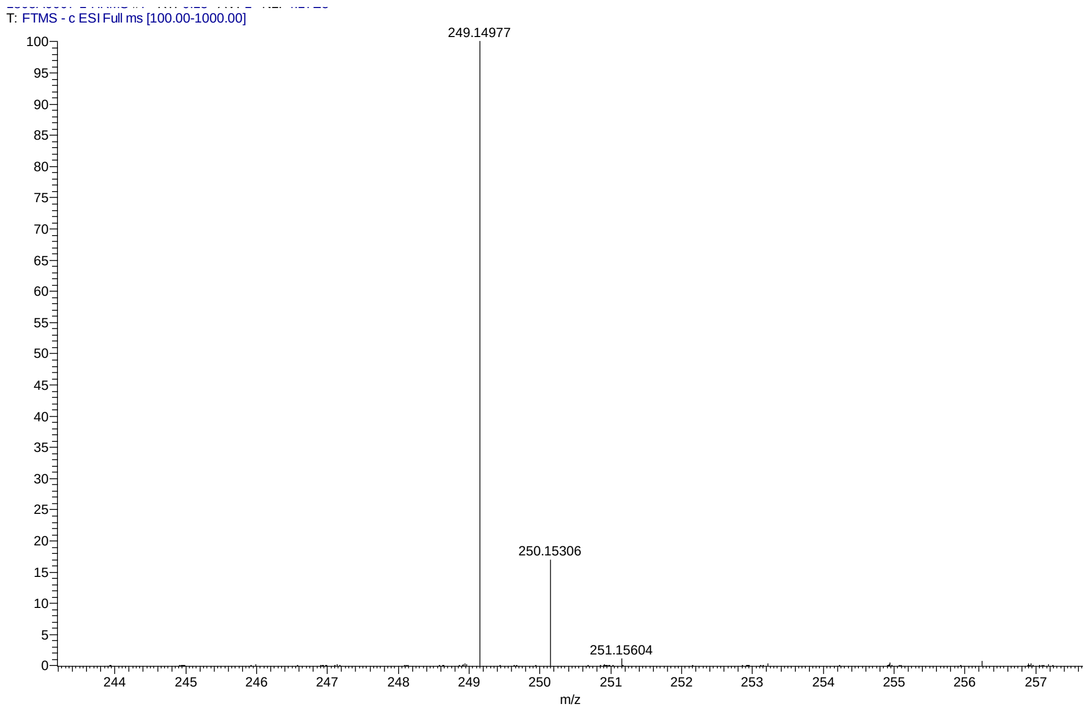

Fig.S37. HRESIMS spectrum of compound **6**

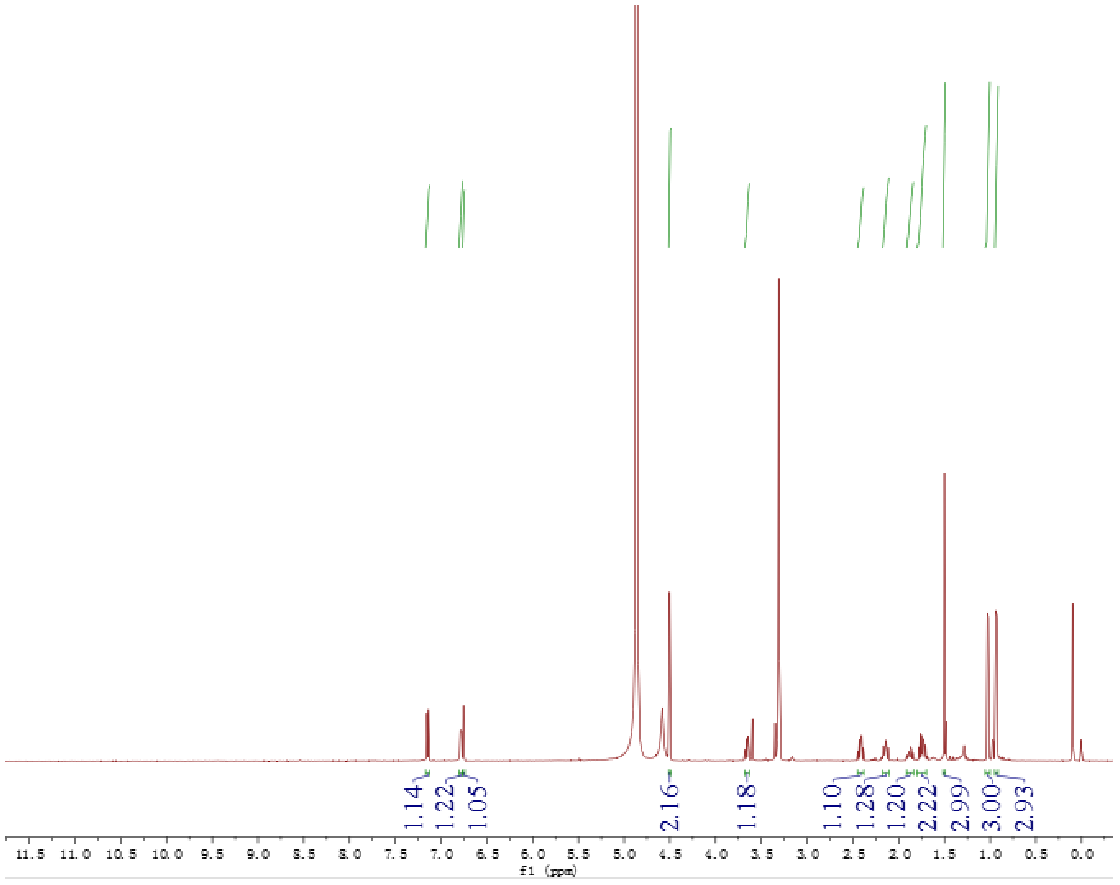

Fig.S38. ^{1}H NMR (500 MHz, Methanol-d_4) spectrum of compound **6**

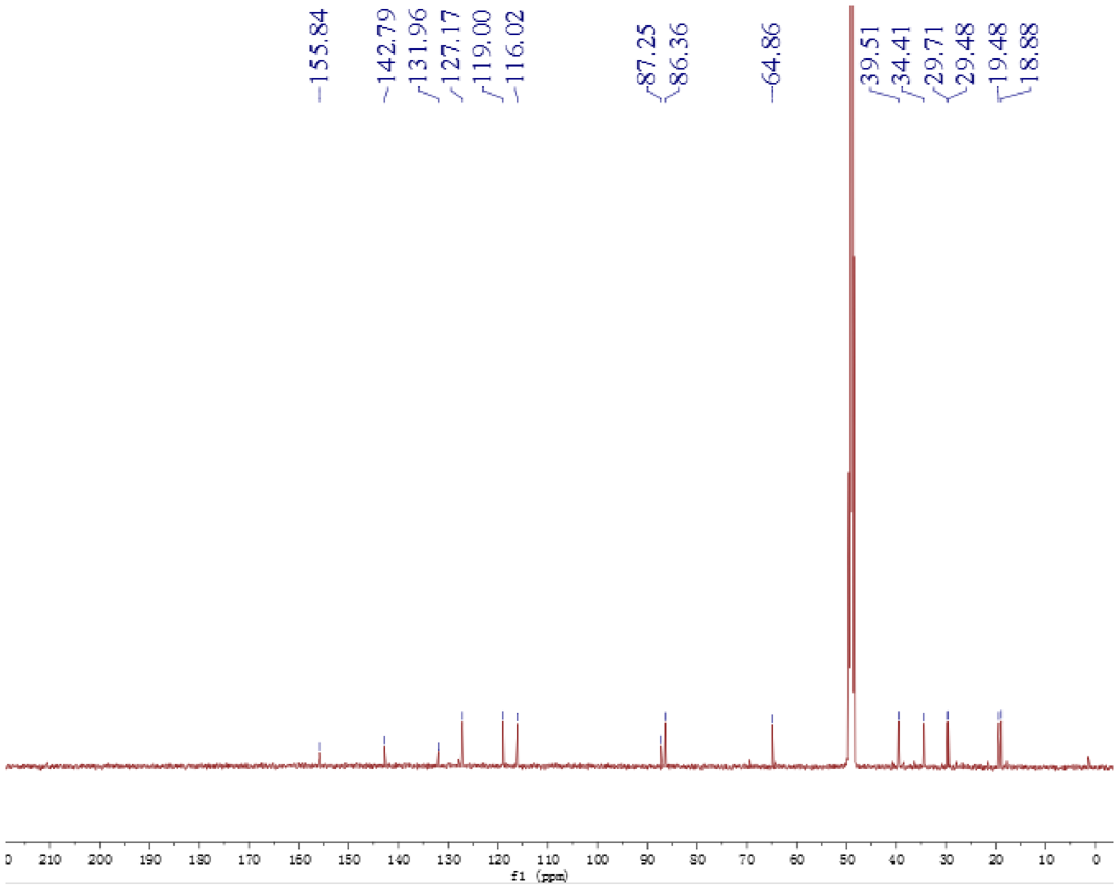

Fig.S39. ^{13}C NMR (125 MHz, Methanol-d_4) spectrum of compound **6**

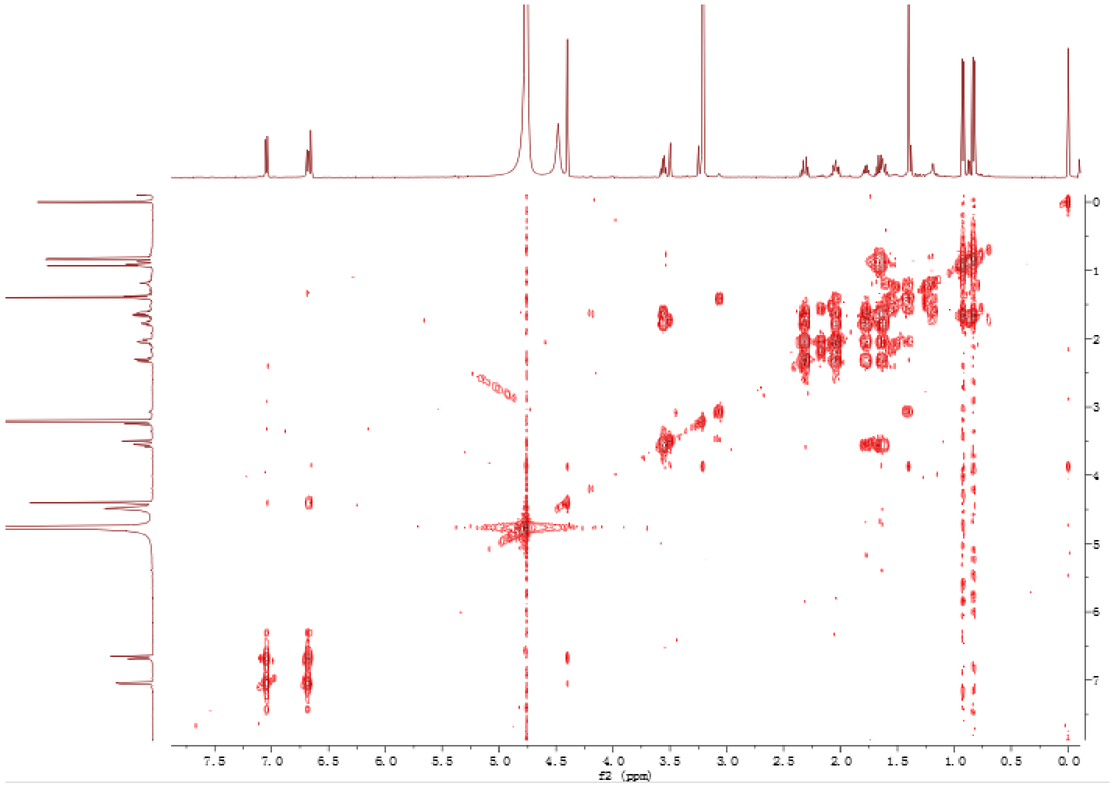

Fig.S40. ^{1}H- ^{1}H COSY (Methanol-d_4) spectrum of compound **6**

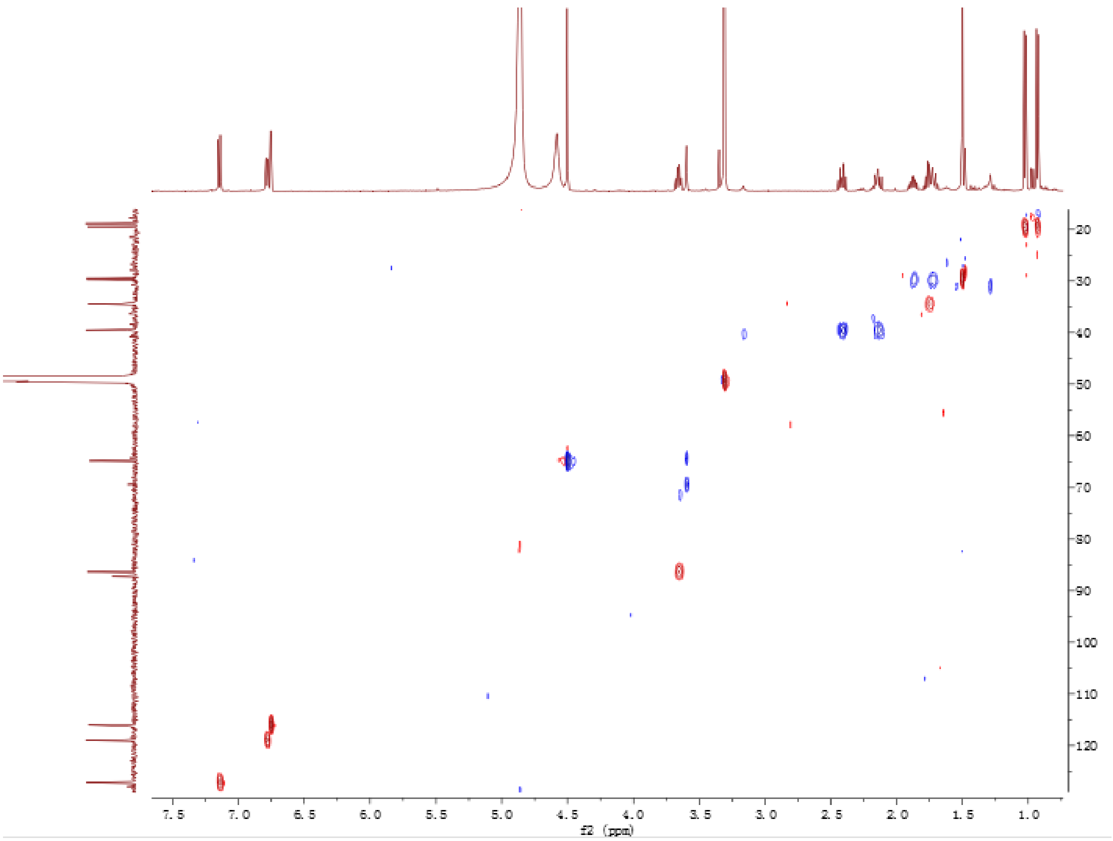

Fig.S41. HSQC (Methanol-d_4) spectrum of compound **6**

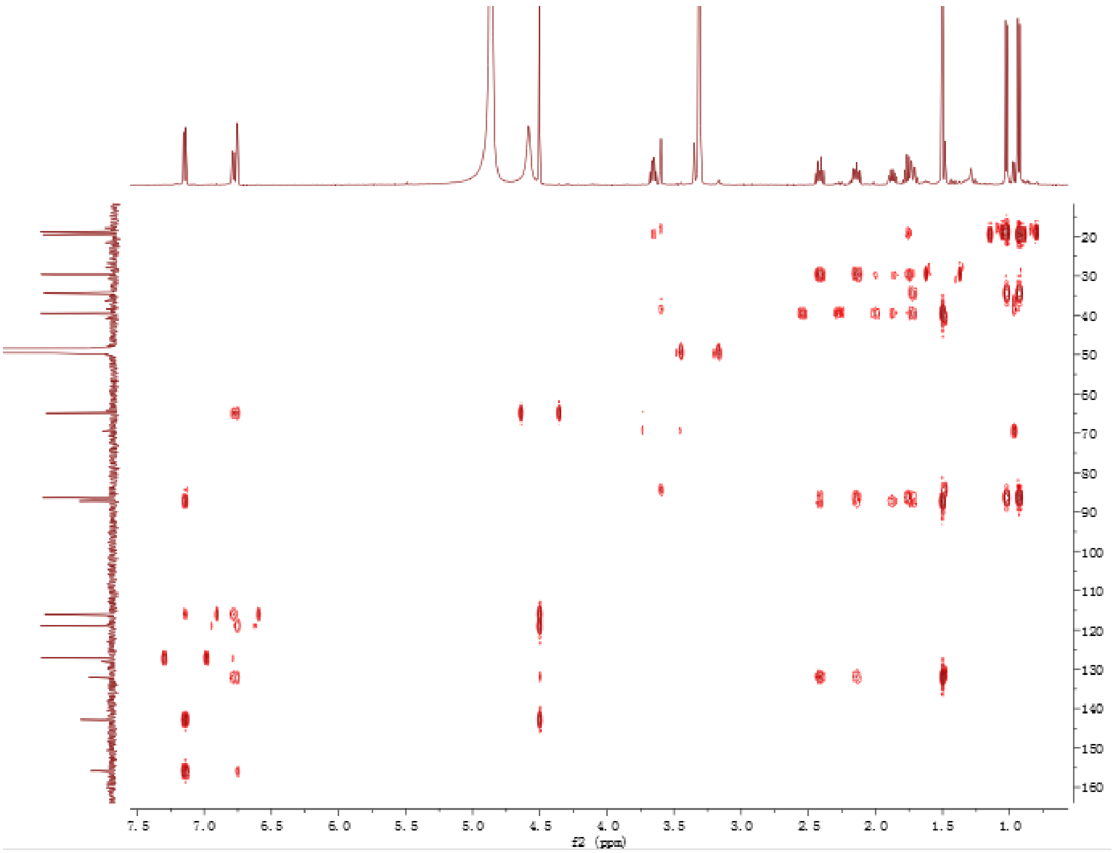

Fig.S42. HMBC (Methanol-d_4) spectrum of compound **6**

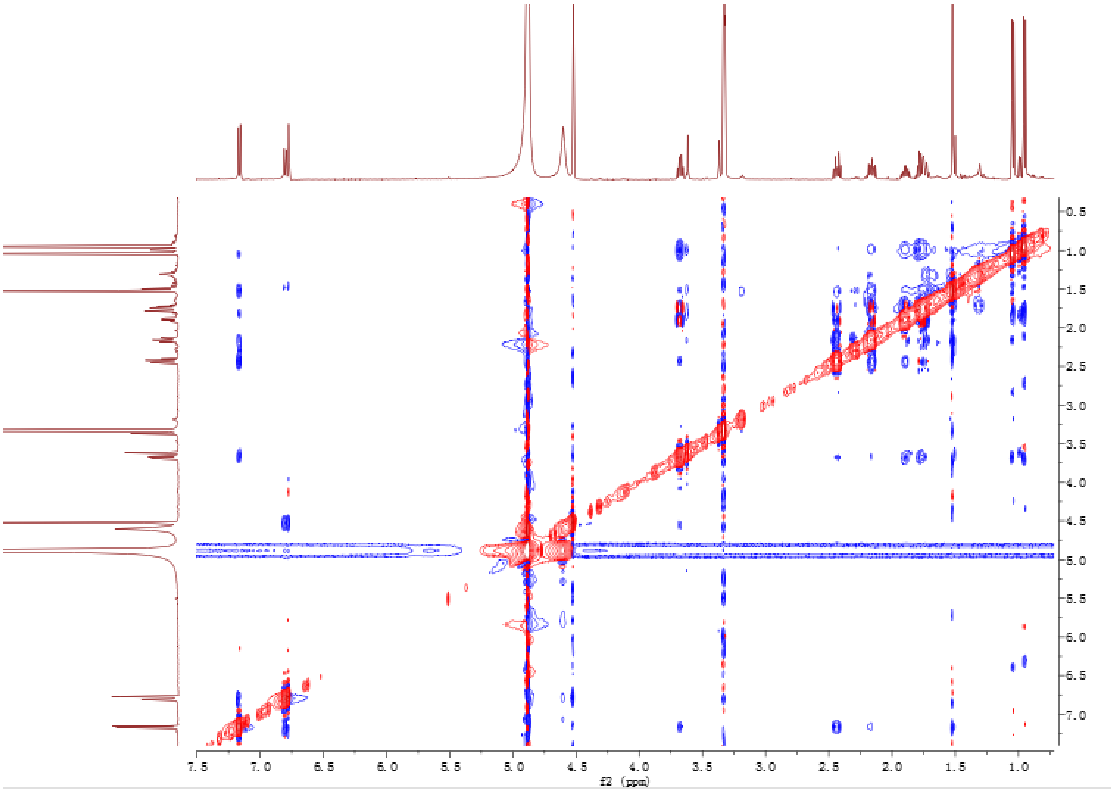

Fig.S43. NOESY (Methanol-d_4) spectrum of compound **6**

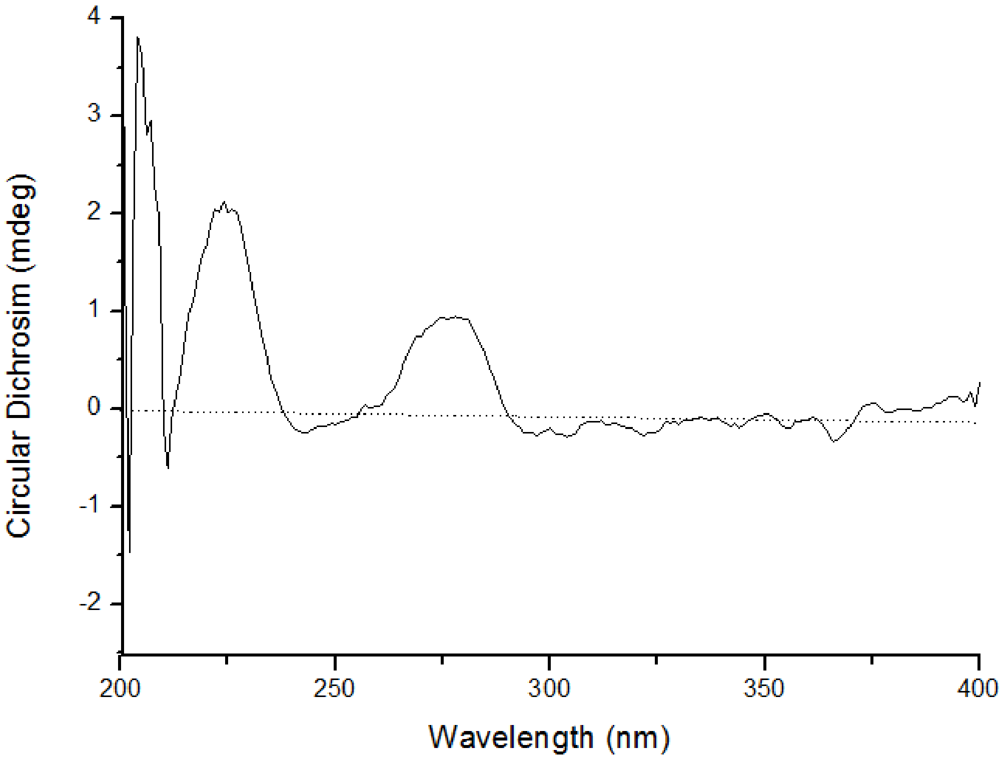

Fig.S44. Experimental ECD spectrum of compound **6**
